# Developing films to support vaccine-hesitant, ethnically diverse parents’ decision-making about the human papillomavirus (HPV) vaccine: a codesign study

**DOI:** 10.1136/bmjopen-2023-079539

**Published:** 2024-09-12

**Authors:** Harriet Fisher, Sarah Denford, Tracey Chantler, Suzanne Audrey, Adam Finn, Huda Hajinur, Matthew Hickman, Sandra Mounier-Jack, Marion Roderick, Leanne Tucker, Julie Yates, Asha Mohamed

**Affiliations:** 1 National Institute for Health Research Health Protection Research Unit (NIHR HPRU) in Behavioural Science and Evaluation (BSE), Bristol Medical School, University of Bristol, Bristol, UK; 2 National Institute for Health Research Health Protection Research Unit (NIHR HPRU) in Vaccinations and Immunisation, London School of Hygiene & Tropical Medicine, London, UK; 3 Schools of Population Health Sciences and of Cellular and Molecular Medicine, University of Bristol, Bristol, UK; 4 Caafi Health Community Interest Company, Bristol, UK; 5 Department of Paediatric Immunology and Infectious Diseases, University Hospitals of Bristol and Weston NHS Foundation Trust, Bristol, UK; 6 Not applicable, Bristol, UK; 7 NHS England South West, Taunton, UK

**Keywords:** PUBLIC HEALTH, Paediatric infectious disease & immunisation, IMMUNOLOGY

## Abstract

**Abstract:**

**Objective:**

To illustrate an evidence-, theory- and person-based approach to codesign the COMMUNICATE films that support parental decision-making about the human papillomavirus (HPV) vaccine for their teenagers.

**Design:**

Codesign study.

**Setting:**

Localities covered by two immunisation teams in London and the south-west of England.

**Methods:**

The intervention planning phase involved combining evidence from a literature review with qualitative interview data to identify barriers and facilitators to HPV vaccine uptake, as well as design features that should be incorporated within the COMMUNICATE films. The intervention development phase involved identifying guiding principles for the COMMUNICATE films, mapping behaviour change techniques onto the behaviour change wheel and codesigning the COMMUNICATE films. Feedback from users informed modifications to maximise acceptability and feasibility and to support behaviour change.

**Results:**

The primary and secondary evidence highlighted important content to include within the COMMUNICATE films: emphasise the benefits of the HPV vaccine, provide transparent information about the safety profile and side effects and emphasise the universality and commonality of HPV infection. A series of scripts were used to guide 4 film shoots to create the content in multiple community languages with 16 participants, including vaccine-hesitant, ethnically diverse parents and professionals. Overall, participants were positive about the films. Potential messengers and ways the films could be distributed, identified by parents, include local social media networks or text messages from general practices. The need for information about the HPV vaccine to be shared by schools ahead of consent being sought was also raised.

**Conclusions:**

By using an integrated approach to intervention development, this study has begun to address the need for an intervention to support vaccine-hesitant, ethnically diverse parents’ decision-making about the HPV vaccination programme. A future study to codesign, implement and evaluate a communication strategy for the COMMUNICATE films is planned.

**STRENGTHS AND LIMITATIONS OF THIS STUDY**

- We involved parents from a range of ethnic backgrounds, including those who are usually under-represented in research, to maximise the acceptability, feasibility and persuasiveness of the COMMUNICATE films.
- A parent contributed as a member of the project team to ensure the parent’s voice was included throughout all stages of the research.
- The budget for the study had been adequately costed to ensure sufficient funding for meaningful involvement, both in terms of payment for public contributors and capacity for the research team.
- We were unable to recruit fathers of teenagers as film participants (although male health professionals were included).
- Not all ethnic groups were represented as film participants, and the COMMUNICATE films developed may not address the information needs of all communities.

## Background

 Human papillomavirus (HPV) is a commonly occurring, self-limiting infection that is primarily transmitted through skin-to-skin contact, including sexual contact. Rarely, persistent infection with high-risk HPV types can lead to the development of cancers affecting both women and men, including the cervix, vulva, vagina, penis, anus and oral cavity. The three HPV vaccines—bivalent, quadrivalent and non-valent—all have proven safety profiles and are efficacious in producing strong immune responses when administered in early adolescence.^1 2^

In England, the universal HPV vaccination programme is offered to young people aged 12–13 years and usually delivered in the school setting. The most recent data for the 2021/2022 HPV vaccination programme showed coverage has fallen below 70% nationally, related to higher levels of school absence, reduced consent form returns and increased vaccine hesitancy in some areas.^3^ Further, there is persistent lower uptake among ethnically diverse populations.^4 5^

Uptake of vaccination programmes can be influenced by multiple interacting forces, which can be population specific and include vaccine hesitancy. Vaccine hesitancy refers to a delay in acceptance or refusal of vaccines despite availability of vaccination services. Vaccine hesitancy is complex and context specific, varying across time, place and vaccines. It is influenced by factors such as complacency, convenience and confidence.^6^

Vaccine hesitancy is recognised as an important contributor to inequalities in uptake of vaccination programmes among minority ethnic groups.^7 8^ Contributors to higher vaccine hesitancy among minority ethnic groups include concerns relating to safety and side effects of vaccines, a lack of trust in the medical profession due to historical discrimination, racist ideology and immoral experimentation on specific racial groups.^8^,^11^ Meaningful engagement and tailored information can support effective vaccine promotion. Engagement and knowledge are facilitated by collaboration with trusted community representatives. To address inequalities in vaccine hesitancy, community-led strategies are required that incorporate meaningful engagement to ensure diverse local voices are heard and codesign programmes that address local concerns to maximise vaccine uptake from the ground up.^12^ This includes health communication interventions that take into account the cultural and community contexts that influence beliefs and decisions about vaccines among different groups.^13^

During the COVID-19 pandemic, the spread of antivaccine information has increased on social media and has contributed to increased levels of vaccine hesitancy.^14^ Further, public perceptions of the importance and safety of the HPV vaccine have reduced.^15^ Additional, and alternative, communication resources and channels about the HPV vaccination programme are needed to reach vaccine-hesitant parents who do not respond to routine communication by school immunisation teams.

### Intervention planning and development

The most recent Medical Research Council and the National Institute for Health Research 2021 guidance advises that the development of complex interventions should systematically draw on the latest evidence and be by guided by appropriate theory.^12^ However, behavioural change interventions are often developed without this.^13^ Calls have also been made for researchers to better report processes and decision-making to address under-reporting and increase clarity of intervention development.^14^

Codesign research approaches involve sharing decision-making and including the expertise of the target users delivering and receiving an intervention.^15^ By incorporating their perspectives, the acceptability, feasibility and practicality of the intervention are addressed and maximised at the intervention development stage.

As part of a wider study, we are developing a targeted, multicomponent intervention to increase parental vaccine confidence in, and adolescent access to, the HPV vaccination programme in England.^16^ The aim of this manuscript is to document, in a transparent manner, the intervention planning and development processes we used to codesign the COMMUNICATE films developed as part of this. The specific objectives are to:

1. Describe the process of involving target users throughout the development of the content for the COMMUNICATE films.
2. Provide information about how their feedback shaped the design of the COMMUNICATE films.

## Methods

### Research setting

The research was undertaken in areas of London (Hackney, Tower Hamlets) and South West of England (City of Bristol), where uptake rates of the HPV vaccination programme are ranked below the national average of 69.6% of the cohort receiving the first dose (61.8% and 68.5%, respectively).^3^

### Patient and public involvement

Patient and public involvement has been integrated throughout the study. Our project team includes a member of the public (LT), ensuring parents perspectives have been captured at all stages. Her role has included attending and contributing to all project meetings and supporting analyses of the interview data. She has also been involved throughout codesign of the films by attending meetings with the creative team, providing feedback on scripts and contributing to creating content during two film shoots.

Consultations regarding recruitment and intervention design were facilitated through parent community organisations in Bristol and London. Resulting changes to the study design include modifications to the participant information sheet, offering a choice of gift voucher and increasing remuneration amount.

Through collaborating with colleagues at Caafi Health (a Community Interest Company aiming to address health inequalities), we have ensured that the voices of population groups who are often under-represented in research and healthcare have been integrated within the study. Meaningful changes to how the research activities have been undertaken include the provision of interpretation support to ensure that people who do not speak English as their first language are included both as research participants and public contributors. Their input regarding inclusion of speaking their native language when developing the content for the films was taken forwards, and filming took place in multiple languages (Arabic, Somali, English) with the support of interpreters.

### Study design

We used the person-based approach for intervention development^17^ in combination with the behaviour change wheel (theory-based, intervention development approach)^18 19^ to develop an appropriate theory-based, evidence-based and person-based framework to underpin the COMMUNICATE films.

**Figure 1:**
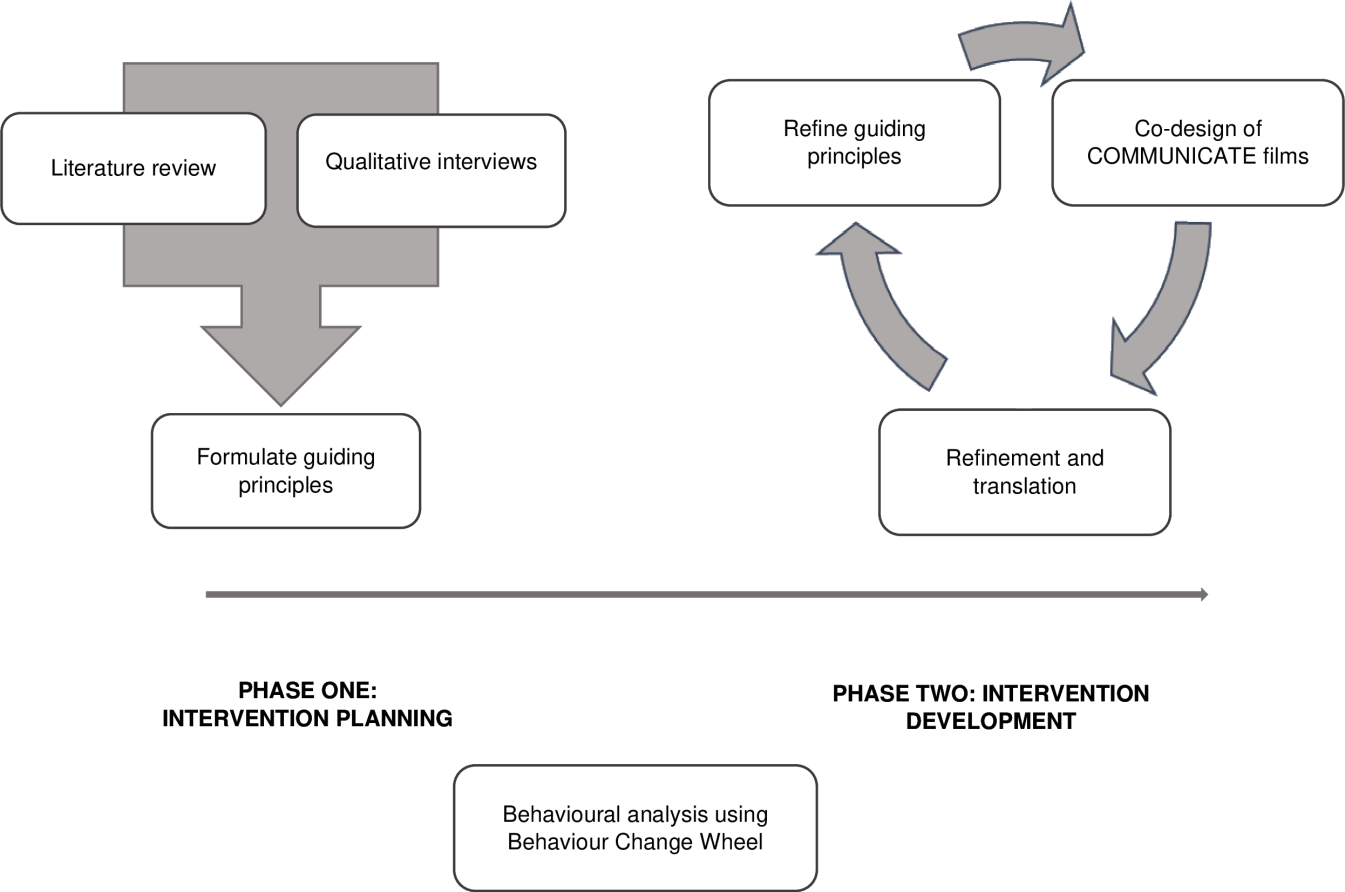
Intervention development overview adapted from the person-based approach.

The intervention planning stage included collating primary and secondary data through a literature review and qualitative interviews, respectively. The intervention development stage involved drawing on this evidence to develop guiding principles, codesign, refinement and translation of the COMMUNICATE films, and a behavioural analysis of content.^17^ These methods were conducted in an iterative manner, with each step informing the next (figure 1)

### Intervention planning stage

#### Literature review

As recommended by the ‘person-based approach’, a literature review was undertaken to identify relevant key behavioural determinants among parents that could prevent young people being vaccinated through the English vaccination programme. Evidence was collated through the literature review, and key papers that examined behavioural issues were also identified by the study researcher (HF) who has extensive knowledge of the literature, (including undertaking systematic reviews relevant to the topic), from previous systematic reviews and handsearching of citations and reference lists.

#### Qualitative interviews

The purpose of the qualitative interviews was to elicit user views relevant to intervention development and codesign of the COMMUNICATE films. Qualitative interviews were undertaken with 29 ethnically diverse, vaccine-hesitant parents. Electronic and paper-based information about the study, targeting parents whose teenage children had not received the HPV vaccine, was sent by school-aged immunisation teams and community organisations within the study areas (City of Bristol, Tower Hamlets and Hackney). Interviews were undertaken by the study researchers (HF, TC and SD) using semistructured topic guides (online supplemental file 1) and took place between April and August 2022 either by telephone or digital platform according to participant preference. Recruitment continued until no new themes were identified during the interviews. Further detail on the methodological approach is provided elsewhere.^20^

Recordings were transcribed verbatim and thematic analysis^21^ was undertaken assisted by NVivo V.12 software. Both inductive and deductive approaches were employed to analyse the content, focusing on the main research question while identifying key information needs emerging from the data. Themes were identified within which similarities and differences were explored.

Analyses of the data focused on identifying key issues relevant to intervention development and codesign of the COMMUNICATE films. These were ‘mechanisms to raise the profile of the HPV vaccination programme’ (‘credible information’ and ‘awareness raising’) and ‘structure of films’.

### Intervention development

#### Behavioural analysis

The purpose of this phase was to undertake a behavioural analysis by systematically identifying and describing intervention components using the behaviour change wheel.^18^ The behaviour change wheel^18^ was selected as it was designed to help researchers link behaviours to inform intervention design. The behaviours related to uptake of the English HPV vaccination programme identified during the intervention planning stage were mapped onto constructs from the behaviour change wheel.^18^ This enabled the researchers to clearly describe the intervention processes and components, including behavioural domains, intervention functions and the behaviour change techniques^19^ to be targeted.

#### Development of guiding principles

The purpose of this phase was to identify key behavioural needs, challenges or issues the intervention needed to address. In the ‘person-based approach’, guiding principles comprise a design objective and intervention features that address the specific behavioural needs of the target audience that were identified during the intervention planning stage and are likely to influence engagement with the intervention. Provisional guiding principles were iteratively developed by the research team and refined as further understanding was gained throughout the study.

### Codesign of the COMMUNICATE films

The next stage of the research involved codesigning the content of the COMMUNICATE films to meet the information needs of parents identified during the intervention planning phase. The study researcher (HF) developed a provisional plan for filming, outlining the key information to include, mechanisms to raise profile of the HPV vaccine and suggested structure of the videos (online supplemental file 2). The plan was informed by the literature review, analyses of interview data with vaccine-hesitant parents and ongoing discussions with key stakeholders (including members of school-aged immunisation teams, parents from ethnically diverse backgrounds, community advocates and academics).

An initial meeting was held with the filmmakers, alongside the project team (HF, SD, TC and LT) and community advocates (AM, HH). The purpose of the meetings was to agree the storyline of the films as a group and provide feedback on the provisional filming plan.

The study researcher (HF) developed initial scripts to guide each of the planned film shoots (online supplemental file 3), which specifically aimed to address information needs raised by vaccine-hesitant parents as part of the wider qualitative study.^20^ Responses within the scripts were informed by a learning resource developed to support healthcare professionals balance arguments and debunk vaccine disinformation (https://jitsuvax.info/). The resource is based on a systematic literature review of 152 scientific articles, a thematic analysis of 2066 antivaccination arguments.^22^ Written feedback on the tone and content of the scripts was sought from the academic research team, alongside a wide range of stakeholders (n=14), including parents, community advocates, immunisation nurses, consultant oncologists and radiologists, and psychologists. This ensured the content was accurate, evidence based and consistent with the best clinical and educational practice.

Four in-person film shoots were organised around the following scenarios confirmed during the intervention planning phase: (1) healthcare professionals and vaccine scientists providing HPV vaccine related information; (2) parents interviewing healthcare professionals to find out key information to inform decision-making; (3) parents of unvaccinated teenagers discussing vaccination with parents of vaccinated teenagers and (iv) a case study with a person who has experienced HPV-related cancer.

Film shoots took place across the research sites at a hospital, community organisation, a university and at the home of a participant. The films shoots involved the filmmaker, members of the research team (HF and SA) and 13 individuals, including a vaccine scientist, healthcare professionals (consultant in gynaecology oncology, a general practitioner and a nurse), a cervical cancer survivor and parents of vaccinated and unvaccinated teenagers.

#### Refinement and translation of the COMMUNICATE films

This phase was conducted to optimise the content of the COMMUNICATE films to meet the needs of the target users. Once the film shoots were completed, the filmmaker edited the footage in line with the guidance provided within the filming plan and scripts. Feedback on the content and style of the preliminary version of the COMMUNICATE films was obtained from the project team (SA, TC, SD, AF, HF, MH, SM-J, MR, LT and JY) with an initial round of revisions made by the filmmakers. Subsequent feedback was obtained on the revised version of the films from key stakeholders (n = 15) (including members of school-aged immunisation teams, parents from ethnically diverse backgrounds, community advocates and academics). Topic guides were developed to elicit their perceptions of the positive and negative aspects of the films, including suggesting or creating new content (online supplemental file 4).

Responses from all participants were collated in a table of changes document. The researchers (HF, TC and SD) held online meetings to agree on modifications to the COMMUNICATE films in line with the ‘person-based approach’ common guiding principles,^17^ and the guiding principles developed specifically for the COMMUNICATE films. This involved considering whether they were likely to impact on behaviour change or be a precursor to behaviour change (eg, acceptability, feasibility, persuasiveness, motivation and engagement). Prioritisation for changes were based on the must have, should have, could have, would-like (MoSCoW) criteria.^23^

## Results

### Intervention planning

#### Literature review

The literature review highlighted key behavioural determinants influencing uptake of the HPV vaccination programme by young people (online supplemental file 5).

Overall, parents often had limited knowledge and misunderstandings about HPV, the vaccine and diseases affecting men and women preventable through vaccination. Content of the future COMMUNICATE films should emphasise the clinical sequelae of HPV, universality of health benefits by gender and the effectiveness of the HPV vaccine and vaccination programme.

Protection offered in relation to prevention of HPV-related diseases acted as a motivator for parents to vaccinate their teenagers, whereas perceptions of low risk of HPV acquisition could negatively influence parental decision-making. Some parents were also concerned about the potential encouragement of sexual activity associated with receiving the HPV vaccine. Framing HPV vaccine messages to focus on cancer prevention, appealing to parents’ responsibility to protect their teenager’s health, rather than sexual transmission, could help to address barriers to uptake related to stigma.

A key information need relates to the rationale and need for vaccination during early adolescence, with some parents delaying vaccination until they felt their teenager would be engaging in sexual relationships. Messaging should emphasise that the HPV vaccine will provide teenagers with protection for when they do become sexually active, rather than accelerating their sexual debut, and that the vaccine is most effective if provided ahead of potential exposure to HPV. The future COMMUNICATE films should also promote the universality and commonality of HPV, emphasising that transmission can still occur even in sexual relationships within the context of marriage.

Parents expressed worries concerning side effects (eg, fertility issues) and safety of the HPV vaccine. Open, transparent evidence-based information in relation to side effects—both minor and serious—is required to overcome distrust in the HPV vaccine. The COMMUNICATE films should also dispel misconceptions in relation to perceptions of serious side effects.

Finally, in schools-based vaccination programmes, a requirement for parental consent and unreturned consent forms act as barriers to uptake (online supplemental file 5).

#### Qualitative interviews

Findings in relation to content to include within the COMMUNICATE films have been reported separately,^20^ and the findings summarised within the literature review (see online supplemental file 5). Of the 29 parents interviewed, the majority were mothers (79%), belonged to a minority ethnic group (88%) and had an adolescent child unvaccinated against HPV (72%) (table 1). A summary of the findings, and illustrative quotations that were expressed concisely and typify responses relating to the themes, is presented in online supplemental file 6.

**Table 1:** Sociodemographic characteristics of interview participants

Interview	Gender	Location	Ethnicity	Gender of vaccine eligible teenager	Vaccination status of child	Time of interview (min)	
1	Female	Bristol	White British	Male	Unvaccinated	50	
2	Male	Bristol	British Asian	Male and female	Unvaccinated	36	
3	Male	London (Hackney)	Black British	Male and female	Vaccinated	41	
4	Male	London (Hackney)	Black British	Male	Unvaccinated	37	
5	Female	Bristol	White British	Female	Unvaccinated	65	
6	Male	London (Hackney)	Black Caribbean	Male	Unvaccinated	41	
7	Male	London (Hackney)	British South Asian	Female	Unvaccinated	57	
8	Male	London (Hackney)	Black American	Male and female	Vaccinated	44	
9	Female	London (Islington)	Black	Male	Unvaccinated	52	
10	Female	Bristol	White British	Male	Unvaccinated	51	
11	Female	Bristol	Somali	Male	Unvaccinated	32	
12	Female	London (Tower Hamlets)	Somali	Male	Unvaccinated	46	
13	Female	London (Tower Hamlets)	Bengali	Male	Unvaccinated	42	
14	Female	London (Tower Hamlets)	British African	Male	Vaccinated	22	
15	Female	Bristol	Somali	Female	Unvaccinated	49	
16	Female	Bristol	African	Male	Vaccinated	41	
17	Female	Birmingham	Somalian	Female	Unvaccinated	Interview not recorded	
18	Female	Bristol	British African	Male and female	Vaccinated	23	
19	Female	Bristol	Bengali	Male	Unvaccinated	36	
20	Female	Bristol	British African	Not provided	Unvaccinated	42	
21	Female	Bristol	Sudanese	Female	Vaccinated	50	
22	Female	Bristol	Somali	Not provided	Unvaccinated	47	
23	Female	London	Black African	Female	Unvaccinated	23	
24	Female	Bristol	Somali	Male and female	Unvaccinated	41	
25	Female	Bristol	White British	Male and female	Unvaccinated	25	
26	Female	Bristol	Somali	Not provided	Vaccinated	40	
27	Female	London	Somali	Not provided	Vaccinated	Interview not recorded	
28	Female	Bristol	African Somali	Female	Unvaccinated	45	
29	Female	Bristol	Somali	Not provided	Unvaccinated	37	

There were several crucial insights from this stage of the intervention planning. Distrust around the safety of vaccines was apparent, with parents referring to misinformation circulating on social media and perceptions that key information about side effects was not explicitly provided within official circulated information. As widely trusted sources of vaccine information, healthcare professionals could play an important role in dispelling misconceptions and providing reassurance to parents. The inclusion of the story of an HPV-related cancer survivor could help increase parents’ motivation to vaccinate their teenage children by highlighting the potential benefit of prevention.

Perceptions that information about the safety and side effects of vaccines was withheld from the public were voiced by participants in this study, alongside requests for open, transparent information. The future COMMUNICATE films, and related HPV messages, should directly address the misconceptions that parents voiced around safety and side effects.

Parents were largely supportive of films to raise awareness of the HPV vaccine. Potential messengers and ways the films could be distributed include: (1) health promotion days; (2) local social media networks and (3) text messages from general practices. The need for information about the HPV vaccine to be shared by schools ahead of consent being sought was also raised.

Representation of film participants (healthcare professionals and parents) from different ethnic groups was felt to be important to ensure relevance of the COMMUNICATE films to the target population and could help normalisation of vaccination within different communities. Further, the future COMMUNICATE films should be developed in different languages to ensure that parents who do not speak English as their first language are not excluded from the decision-making process.

### Intervention development

#### Behavioural analysis

The proposed COMMUNICATE film employs four intervention functions (enablement, education, persuasion and environmental restructuring), which are enacted by six behavioural change techniques (‘instruction on how to perform a behaviour’, ‘information about health consequences’, ‘anticipated regrets’, ‘generalisation of target behaviour’, ‘pros and cons’ and ‘restructuring the social environment’) (table 2).

**Table 2:** Behavioural analysis of COMMUNICATE films using the behaviour change wheel and the behaviour change technique taxonomy

Target behaviour	Barriers to target behaviour	Intervention strategy	Intervention function^18^	Behavioural change technique^32^
Increase uptake of the HPV vaccine	Low levels of understanding about the HPV vaccine. Limited perceptions of protection offered by the HPV vaccine. Misunderstanding around the potential of developing serious side effects. Perceptions of low risk of exposure to HPV due to sexual transmissibility. Lack of parental engagement with consent procedures.	Communication materials should provide information in a tone appropriate to parents about: Protection offered from HPV-related diseases (cancers, genital warts). Length of immunity. Effectiveness of vaccination programme. Universality of health benefits by gender. Persuasive content of films highlighting benefits to increase motivation to be vaccinated (eg, case study of HPV-related cancer survivor).Provide reassurance to parents about the safety profile of the vaccine by providing clear and transparent information about mild and serious side effects.Include messages emphasising the universality and commonality of HPV and clear explanation of why the HPV vaccine is offered during adolescence.Representation of film participants (both healthcare professionals and parents) from different communities.Availability of films in different languages to ensure understanding among parents who do not speak English as their first language.Provide clear instructions about how to get the HPV vaccine routinely both within and outside the schools-based HPV vaccination programme	Enablement (increasing means/reducing barriers to increase capability or opportunity).Education (increasing knowledge or understanding).Persuasion (using communication to induce positive or negative feelings or stimulate action).Environmental restructuring (changing the physical or social contact).	4.1 Instruction on how to perform a behaviour.5.1 Information about health consequences.5.5 Anticipated regret.8.6 Generalisation of target behaviour.9.2 Pros and cons.12.2 Restructuring the social environment.

HPVhuman papillomavirus

#### Development of the guiding principles

The guiding principles, and the key features of the intervention that will help these be achieved, are provided (table 3).

**Table 3:** Guiding principles for the COMMUNICATE films

Design objectives that address each key issue	Key intervention features relevant to each design objective
Increase parents’ motivation for their adolescent child to be vaccinated	Highlight potential consequence of HPV infection (eg, types of cancer). Persuasive content through case study of HPV-related cancer survivor.
Reassure parents for their adolescent child to be vaccinated	Provide transparent, evidence-based information in relation to side effects—both minor (eg, headaches, achy arm, fever) and serious (eg, anaphylaxis, 1 in a million). Deliver evidence-based information in relation to the safety profile of the HPV vaccine (eg, rigour of clinical trials, vaccine safety monitoring data). Dispel misconceptions in relation to perceptions of serious side effects (eg, fertility, cancer) by including parents asking questions to healthcare professionals. Normalise parental concerns and perceptions of potential for long-term harm (eg, include parents discussing their concerns about the HPV vaccine). Representation of trusted healthcare professionals and vaccine scientists.
Overcome stigma of being vaccinated against a sexually transmitted infection	Provide open and transparent information about routes of transmission of HPV. Promote universality and commonality of HPV—everyone is at risk (including sexual relationships within a marriage). Protection for when the adolescent becomes sexually active, rather than anticipating their sexual debut. Representation of film participants (both healthcare professionals and parents) from different communities.
Engage parents in decision-making and the consent process	Provide clear instructions about how to get the HPV vaccine routinely through schools-based HPV vaccination programme, acknowledging geographic differences in delivery. Include information on availability of the HPV vaccine outside of the schools-based programme (eg, community catch up clinics by school-aged immunisation teams, general practice, eligibility by age).
Be delivered flexibly to meet needs of target population	Available in different languages to ensure reach to families who do not speak English as their first language. Communication materials to be available prior to invitation to HPV vaccination programme to ensure benefit for all families. Communication materials to be targeted to parents of unvaccinated adolescent children. Materials can be distributed through different mechanisms of delivery (eg, images for newsletters, short clips for social media, tool for/discussion with parents).

HPVhuman papillomavirus

In brief, the COMMUNICATE films aim to: (1) increase parents’ motivation for their teenager to be vaccinated; (2) provided reassurance for parents to have their teenager vaccinated; (3) overcome stigma of providing consent for a vaccine that protects against a sexually transmitted infection; (4) engage parents in decision-making and consent process and (5) be delivered flexibly to meet the needs of the target population.

#### Codesign of the COMMUNICATE films

The following key themes for content were identified: (1) protection offered by the HPV vaccine; (2) sexual transmission and (3) safety and side effects.^20^ Providing parents with clear instructions on how to get their teenager vaccinated was also viewed as important.

The translation of the films to multiple community languages using subtitles or voices overs to ensure reach to the target population was discussed. Community advocates suggested it would be more authentic for some of the participants to be speaking in their native language within the films. This suggestion was taken forwards and incorporated within a film shoot involving parents of unvaccinated teenagers.

Film participants (including parents and healthcare professionals) were ethnically diverse (White British (n=5), British Asian (n=2), Black African (n=6)) taking into account the feedback the research team received regarding the importance of representing different communities within the communication materials.

#### Refinement and translation of the COMMUNICATE films

Initially, the COMMUNICATE films were presented as one main film approximately 6 min in length presenting information around the key themes identified in the earlier phases of the intervention planning. A separate film dedicated to sharing the story of a cervical cancer survivor was also created.

Positive feedback on the main film from the project team included the use of graphics and creative shots, and the inclusion of footage in multiple languages. Critical feedback suggested overall the story of the main film was difficult to follow, in part because of the ordering of the content and as the footage appeared to cut quickly between participants. The project team also questioned whether important content had been excluded as part of the editing process. The main feedback on the story of the cervical cancer survivor was that the film was presented as two separate stories (having cancer vs getting the vaccine) which needed to be linked together.

In response to the feedback, the lead researcher (HF) reviewed the unedited footage from each of the shoots and suggested to the filmmaker additional footage to include. The main film was subsequently broken down into a series of subfilms, each focusing on one of the key themes proposed (eg, safety and side effects). Additional footage relating to the cervical cancer survivors’ discussion on fertility, her own experience of stigma following diagnosis and people’s perceptions of her sexual behaviour were added to this film to connect the story.

Further minor changes to the films included: (1) correcting typos or changing words to improve clarity of meaning within the graphics provided; (2) clarifying the role of each of the film participants by increasing the size and length of presentation of subheadings and (3) including a signposting slide at the end of each film with relevant sources for further information about the HPV vaccine and how parents could get their teenager vaccinated.

#### Summary of feedback from ethnically diverse parents and stakeholders

Feedback was obtained from seven parents (White British (n=2), Black African (n=3) and British Asian (n=2)) and stakeholders (n=7) (including graphic designer, healthcare professionals and biomedical scientist).

Overall, parents and stakeholders were positive about the films and suggested they would be beneficial in helping support parents’ decision-making about the HPV vaccine for their teenager. Positive feedback included being able to select topics of interest through the series of films, the ethnic diversity of film participants, use of multiple languages and the inclusion of a personal story to persuade parents to vaccinate their teenagers.

There was mixed feedback on the length of the films, with some feedback indicating that the films should be shortened to improve the likelihood that parents would engage with the content. Although it was acknowledged that some parents would likely disengage from the films, it was decided that they would be more appealing to the target population: vaccine-hesitant parents with information needs.

Further changes included: (1) edits of film footage to ensure clinical accuracy and avoid misunderstanding of messaging, (2) adding additional content as visuals to either confirm messages or providing key information not discussed by participants; (3) including a ‘key messages’ slide at the end of each film to reinforce information provided and (4) amending a graphic to place images of viruses around throat and genital areas, rather than the chest. Two of the films were subsequently re-edited and combined as one film to ensure succinctness following feedback highlighting repetition of content around safety and side effects.

#### Translation

Once the films were finalised, transcripts of the content of each of the films were created which were translated by professional translators into five key community languages (Arabic, Bengali, Polish, Urdu and Somali). Voice-overs were provided by native speakers and edited onto the film.

The final package for the COMMUNICATE films comprises four subfilms entitled: ‘protecting my teenager’, ‘Penny’s story’, ‘safety and side effects’ and ‘the HPV vaccine and my teenager’. We intend to make the final product available in the public domain shortly.

## Discussion

The aim of this manuscript was to illustrate how a systematic, theory, evidence and person-based approach^17^ was used to codesign the COMMUNICATE films. The films are intended to increase parents’ motivation to protect their adolescent child by highlighting the benefits of protection against cancer, to provide accurate convincing information in relation to the excellent safety profile and to emphasise the importance of providing HPV vaccine at the recommended age, all alongside communicating the universality and commonality of HPV infection. The COMMUNICATE films are tailored to meet the information needs of ethnically diverse parents in the context of the English HPV vaccination programme. Although developed in two regions, the COMMUNICATE films are likely to be relevant to other ethnically diverse communities in other regions of England. The films could be used as a tool by healthcare professionals to enhance communication about the HPV vaccination programme.

Through our qualitative research and ongoing patient and public involvement, we have identified potential messengers and ways the COMMUNICATE films could be distributed, including text messages from general practices, health promotion days and using local social media networks. We now plan to undertake a future study working alongside key stakeholders and parents to refine the practicalities of how the films could be distributed, support implementation activities and gather evidence in relation to changes to uptake of the HPV vaccination programme. Together with an educational resource we have coproduced with young people^24^ (available from: https://pshe-association.org.uk/resource/educate-hpv-vaccine), these could contribute to a system-wide approach to improving communication about, and increasing uptake of, adolescent vaccination programmes.

Elsewhere, there are approaches to improving communication with parents which show promise. In a US study, a communication experiment aiming to address parents’ concerns through short videos was shown to lead to higher confidence and lower hesitancy for the teenagers to be vaccinated against HPV.^25^ In 2017, Danish health authorities launched a media campaign, which included a series of educational videos, to increase health literacy and restore public confidence in response to negative media reports related to the safety of the HPV vaccine. The campaign was associated with restoration of uptake of their HPV vaccination programme to its baseline level.^26^ Evidence from a randomised controlled trial undertaken in a healthcare setting suggested uptake could be increased through motivational interviewing by healthcare professionals and supplementary information for HPV vaccine-hesitant parents.^27 28^ This study addresses the need for tailored communication materials to meet the needs of ethnically diverse, vaccine-hesitant parents in the context of the English HPV vaccination programme.

### Key learning

In recent years, there has been an increase in the use of codesign research methodologies that involve sharing decision-making and include the expertise of the target users for intervention.^29^ The insights of the target population and public contributors were crucial in developing content and selecting design features of the COMMUNICATE films to meet the needs of vaccine-hesitant, ethnically diverse parents. We are very grateful to have had the support of colleagues at Caafi Health Community Interest Company (an organisation aiming to reduce health inequalities) throughout the study. Their invaluable support has ensured that we have been able to include population groups who are under-represented in research within our study.

We aimed to be as inclusive and flexible as possible to accommodate and support the needs of the contributors to the study. This included practical measures such as provision of translator support and accommodating needs of parents with young children during film shoots, in addition to payment for their time and coauthorship of research publications.

This project was planned by taking into account the timescales required to undertake engagement activities, with a sufficient budget to support this. However, barriers to the substantial involvement of target users using codesign methodologies will persist where there are limited resources and timeframes for projects.^30^ In response to this, there has been increasing emphasis on developing new ways to rapidly develop effective interventions and messaging by integrating co-production methods with experimental, quasiexperimental and real-world evaluation, which could be helpful where timescales and barriers are restrained.^31^

### Strengths and limitations

A strength of the study is the involvement of the parents from a range of ethnic backgrounds, including those who are usually under-represented in research. Their involvement may help improve the acceptability, feasibility and persuasiveness of the COMMUNICATE films. We also involved a parent as a member of the project team to ensure the parent’s voice was included throughout all stages of the research. The budget for the study had been adequately costed to ensure sufficient funding for meaningful involvement, both in terms of payment for public contributors and capacity for the research team.

There are some limitations to the study. We included the perspectives of fathers within our qualitative study. However, we were unable to recruit fathers of teenagers as film participants (although male health professionals were included). Although film participants were ethnically diverse, not all ethnic groups were represented (eg, Eastern European, Gypsy, Traveller and Roma) and the COMMUNICATE films developed may not address the information needs of all communities.

## Conclusion

This study has begun to address the need for a rigorously developed, theory-based intervention tailored to meet vaccine-hesitant, ethnically diverse parents’ information needs about the English HPV vaccination programme. By working closely with parents, the content of the COMMUNICATE films was developed to meet the needs of target users. A future study to codesign, implement and evaluate a communication strategy for the COMMUNICATE films is planned.

## supplementary material

10.1136/bmjopen-2023-079539 online supplemental file 1

10.1136/bmjopen-2023-079539 online supplemental file 2

10.1136/bmjopen-2023-079539 online supplemental file 3

10.1136/bmjopen-2023-079539 online supplemental file 4

10.1136/bmjopen-2023-079539 online supplemental file 5

10.1136/bmjopen-2023-079539 online supplemental file 6

## Data Availability

Data are available on reasonable request.
