# Supplementary material for: Developing films to support vaccine-hesitant, ethnically diverse parents’ decision-making about the human papillomavirus (HPV) vaccine: a codesign study
Source: BMJ Open. 2024 Sep 12;14(9):e079539. doi: 10.1136/bmjopen-2023-079539 (PMC11409246; doi:10.1136/bmjopen-2023-079539)
Supplement: online supplemental file 2 [file bmjopen-14-9-s002.pdf]

**PURPOSE OF DOCUMENT**

This document outlines an overview of the proposed:

1. Key content to include in co-developed communication materials
2. Mechanisms to raise profile of the HPV vaccine
3. Structure of the videos.

The initial plan has been informed by analyses of interview data with parents undertaken as part of the study and discussions with key professionals. Key action points to incorporate during development of communication materials are highlighted within the document.

More detailed scripts/plans will be developed prior to each filming shoot. Further analyses/ discussions/ meetings will inform co-design of an intervention plan to improve parents' confidence in, and adolescents' access to, the HPV vaccination programme.

**Table 1. Key content to include in communication materials**

| Theme | Action point |
|---|---|
| ***i. Levels of awareness and knowledge*** | |
| - The majority of parents appeared to have limited knowledge about the benefits and risks of their adolescent children receiving the HPV vaccine, with many parents confirming that this was influential to their adolescent child not receiving the HPV vaccine. | **Content of the communication materials needs to provide information to address parents' information needs and ensure informed decision making.** |
| - Some parents whose adolescent children had received the HPV vaccine had unanswered questions. | **Communication materials to be available prior to invitation to HPV vaccination programme to ensure benefit for all families.** |
| - Provision of information about the HPV vaccination programme in English language only contributed to a lack of understanding by some parents interviewed. It was also acknowledged to be influential within the wider community. | **Development of communication materials in multiple community languages.** |
| ***ii. Protection offered*** | |
| - All parents reported that their adolescent children had received vaccinations when they were babies under one year old, but some had refused, or delayed, vaccinations offered to young children (e.g. MMR).<br><br>- Among some parents, vaccinations offered during adolescence were perceived as optional and there was hesitance as to why vaccinations would be necessary as adolescents were perceived as 'healthy'. | **Content of communication materials should include:**<br>**- Protection offered from HPV-related diseases (cancers, pre-cancerous changes, genital warts)**<br>**- Effectiveness of vaccination programme (e.g. discussion of results of major studies)**<br>**- Length of immunity**<br>**- Clear explanation of why the HPV vaccine is offered during adolescence.** |
| - Some parents were more cautious in providing consent for their adolescent boys as a result of the recent expansion to a universal HPV vaccination programme. Other parents were unclear as to how adolescent boys could | **HPV vaccine messages should stress universality of health benefits by gender.** |

| | |
|---|---|
| benefit from the HPV vaccine, or whether it would enhance protection to adolescent girls. | **Include male patient affected by HPV-related disease as participant in video to highlight both genders can be affected.** |
| - A few parents highlighted the need for evidence of need in terms of disease burden and effectiveness of the HPV vaccine in reducing associated illnesses. Similarly, a few parents suggested presenting statistics in relation to the occurrence of disease or number to treat. | **Explore with creative team and parents during workshops ways to include information in relation to HPV disease burden and number to treat (e.g. infographics, animations, spoken words).** |
| ***iii. Safety and side effects*** | |
| - All parents highlighted that information around the potential for harm from the HPV vaccine was an important part of their decision-making.<br><br>- Many parents (correctly) identified that the HPV vaccination leaflet does not provide information about side-effects. This appeared to contribute to distrust in relation to why they should have their child vaccinated.<br><br>- Most parents acknowledged that, as with all medicines, there would be the potential for side-effects. Minor side-effects that would last for a short period did not appear to be a major cause for concern or reason not to vaccinate. | **Provide transparent, evidence-based information in relation to side-effects – both minor and serious – within the communication materials.** |
| - A few parents felt reassured that the HPV vaccine did not present risk in terms of side-effects because they had spoken to friends/family whose children had already been vaccinated without issue. | **Include as film scenario parents with vaccinated adolescent children reassuring other parents by discussing their children's experience of side-effects.** |
| - Many parents discussed major concerns in relation to the potential for serious side-effects from their child having the HPV vaccine. Often parents cited the potential for serious, long-term harm, but did not articulate specific health conditions that were attributable.<br><br>- Specific side-effects of the HPV vaccine mentioned by other parents include impact on fertility, causing cancer, developmental issues, and allergic reactions. | **Communication materials should dispel misconceptions in relation to perceptions of serious side-effects.** |

| | |
|---|---|
| - Parents wanted to feel reassured around the safety and side effects of the vaccine in order to support positive decision-making.<br><br>- Reassurance could also be provided through information about the research processes for developing the vaccine, safety measures in place if a child has a reaction following vaccination, and length of time the vaccine has been available. Some parents whose children have long-term health conditions valued information related to contradictions for the HPV vaccine. | **Explore different ways of presenting information with the creative team and parents during workshops.**<br><br>**Provide information about vaccine development from a vaccine scientist and other safety measures.** |
| ***iv. Sexual transmission*** | |
| - Most parents were aware that the HPV vaccine protected against a sexually transmitted infection. However, some parents were unclear how sexual activity could relate to the development of cancer. | **Content of communication materials should address clinical sequelae of HPV.** |
| - Among parents that were aware that HPV was sexually transmitted, there was variation in relation to the extent to which this was influential in decision-making. However, parents felt this information should be provided explicitly in communication materials. | **Provide open and transparent information about routes of transmission of HPV.** |
| - Some parents there appeared to be moral undertones which emphasised providing education to their child to not engage in 'risky' sexual behaviours, rather than relying on the HPV vaccine for protection. | **Messages should promote universality and commonality of HPV – everyone is at risk (including sexual relationships within a marriage).**<br><br>**Ensure representation of film participants from different communities to increase relevance of vaccination (parents and healthcare professionals).**<br><br>**Communication materials should stress importance of vaccination ahead of sexual debut to ensure optimal protection.** |
| - Where parents had lower perceptions of need this predominantly related to the age the HPV vaccine is routinely offered and whether they anticipated that their adolescent child was likely to be at risk of acquiring HPV. | **Include information on availability of the HPV outside of the schools-based programme (may need to address regional differences in GP commissioning).** |

| | |
|---|---|
| - Some parents felt the age the HPV vaccine is appropriate to ensure protection ahead of sexual debut. Other parents could not reconcile this or view an urgency for the vaccination. In these cases, parents preferred for the HPV vaccine to be delayed until their adolescent child was older or sexually active.<br><br>- Some parents felt unable to acknowledge that their child could be sexually active, and therefore at risk of acquiring HPV. This related both to their cultural norms and also perceptions of their child's development/emotional maturity. Other parents were able to balance their own expectations of sexual behaviours with perceptions of intergenerational changes around sexual behaviours within their community.<br><br>- One parent commented that her adolescent sons would not be at risk as it was not within their faith to engage in sexual activity with other males. | **Messages should promote universality and commonality of HPV – everyone is at risk.**<br><br>**Messaging to emphasise that the vaccine offers protection for when the adolescent becomes sexually active, rather than anticipating their sexual debut.**<br><br>**Ensure different communities are represented as participants within the films.** |
| - Some parents reported they had, or would, discuss the sexual transmission of HPV with their adolescent child, with various levels of comfort.<br><br>- Some parents indicated that sex remained a 'taboo' or stigmatised subject within their communities. This inhibited open discussions both with the wider community, and within their family, about the need for the vaccine and decision-making. | **Develop culturally sensitive communication materials which could be used as a tool for parents to discuss the HPV vaccine with their adolescent child.** |

**Table 2. Mechanisms to raise the profile of the HPV vaccination programme**

| Theme | Action point |
|---|---|
| ***i. Credible sources of information*** | |
| - Healthcare professionals with the relevant specialist knowledge were considered to be the most trusted source of information about the HPV vaccine. General practitioners and nurses were frequently referred to, as well as consultants involved in the treatment of HPV-related disease. | **Ensure representation of healthcare professionals from different specialities within communication materials (e.g. GPs, oncologists, nurses).** |
| - A few parents suggested vaccine scientists or immunologists could provide information about the development of the vaccine. | **Seek representation of vaccine scientists in communication materials.** |
| - Many parents discussed (sense checked) the HPV vaccine within their social networks. A few parents indicated they felt judged by other parents for their decision not to have their adolescent child vaccinated. | **Ensure parents from different communities are included as participants within communication materials.** |
| - A few parents felt reassured that the HPV vaccine did not present risk in terms of side-effects because they had spoken to friends/family whose children had already been vaccinated without issue. | **Include as film scenario parents with vaccinated adolescent children reassuring other parents by discussing their children's experience of side-effects.** |
| - Some parents discussed the importance of engaging with faith leaders (e.g. pastors, Iman) to raise the profile of the HPV vaccine. However, more often parents considered that the information should be delivered by healthcare professionals. | **Ensure representation of healthcare professionals from different specialities within communication materials (e.g. GPs, oncologists).** |
| - Online sources were also cited as an important way to verify, or gather information, about the HPV vaccine. These include the NHS website, their social media accounts and YouTube. Some parents indicated they were unsure of the credibility of the information. | **Provide advice on how to identify vaccine misinformation within communication materials.** |

| | |
|---|---|
| - One parent suggested that because of their cultural beliefs that young people would be better placed to promote the HPV vaccine than parents. | |
| ***ii. Mechanisms to improve awareness*** | |
| - Information about the HPV vaccination programme is routinely provided in English language by email or letter from the school on behalf of the immunisation team ahead of the scheduled vaccination session.<br><br>- There was variation in the extent to which this appeared to address parents' information needs. Some parents felt the information was sufficient to inform their decision-making. However, other parents felt the information was insufficient and reported undertaking their own research to address their information needs.<br><br>- Language barriers prevented some parents being able to understand the information received.<br><br>- Despite having vaccine-eligible adolescent children, some parents reported not receiving any information about the HPV vaccination programme. | **Ensure communication materials are available in different languages** |
| - Almost all parents were supportive of videos to share information about the HPV vaccine, but would need to be easily accessible to ensure engagement.<br><br>- Other ways of raising awareness of the HPV vaccination programme included: (i) text messages from General Practice or schools; (ii) face-to-face interactions at community health events; (iii) social media campaigns; (iv) providing information in the Red book. | **Communication materials to be developed which can be supported by different mechanisms of delivery (e.g. images for newsletters, short clips for social media).** |

**Table 3. Structure of videos**

| ***Theme*** | ***Action point*** |
|---|---|
| ***Balancing risk and benefits*** | |
| - Many parents expressed the need for balanced information on the pros and cons of the HPV vaccine to inform decision-making.<br><br>- Some parents suggested healthcare professionals withheld important information in relation to side-effects to ensure high uptake of the vaccination programme. | **Content to provide open and transparent information in relation to both the risks and benefits of the HPV vaccine**<br><br>**Provide guidance on how to assess for 'fake news' or misinformation** |
| ***Presenting risks and benefits*** | |
| - A few parents suggested presenting the statistics (e.g. death rates, side-effects, allergic reactions, effectiveness of vaccination programme) through infographics as an acceptable way of proving this information. | **Explore different ways of presenting information with the creative team and parents during workshops.** |
| ***Scenarios for film shoots*** | |
| - Healthcare professionals with the relevant specialist knowledge were considered to be the most trusted source of information about the HPV vaccine. General practitioners were frequently referred to, as well as consultants involved in the treatment of HPV-related disease. | **Immunisation nurses addressing parents' questions –**<br><br>**Oncology and Radiology consultants**<br><br>**GP and interview/PPI parents (Bristol)** |
| - There was strong agreement from parents that a personal testimony from a person who has experienced HPV-related disease would convey the importance of why their child should be vaccinated. | **Patient story - two patients – older/younger, male/female, different ethnic groups, with parent** |
| - Parents were asked whether they felt faith leaders could advocate for HPV vaccination within the communication materials. Most parents felt it was more appropriate for information to be conveyed by healthcare professionals who had the specialist knowledge. | **Ensure different specialities of healthcare professionals are involved as film participants.** |
| - Some parents felt it would be helpful to include parents discussing their choice about to have the adolescent child vaccinated and whether their adolescent | **Parents discussing vaccination** |

| | |
|---|---|
| child had experienced side effects or not. A few parents did not value this scenario, indicating they would prefer information directly from healthcare professionals. | |
| - A few parents mentioned including vaccine scientists to provide reassurance by explaining the research processes involved in developing the HPV vaccine. | **Interview with vaccine scientist – could include discussion of press 'headlines' (e.g.** HPV is making cervical cancer a 'thing of the past': Jabs prevents nearly 90% of cases \| Daily Mail Online) |
| ***Other*** | |
| - Importance of correct tone of communication materials for target populations. The information should avoid being childish, scaremongering, or appearing too 'professional'. | **Tone of materials should be appropriate for parents and aim to enable informed decision-making.** |
| - A few parents highlighted that the videos should not be too long as this could be off putting. | **Ensure content of communication materials is succinct.** |
| - Some parents discussed the importance of representation from their own, or different, communities. | **Ensure film participants represent different ethnic groups and faiths.** |
| - A few parents discussed that communication materials (e.g. videos) could be used as a tool for discussing the HPV vaccine with their adolescent children. | **Develop content appropriate for adolescents and parents to watch together OR provide young people with EDUCATE videos alongside parent videos.** |
