# Supplementary material for: Developing films to support vaccine-hesitant, ethnically diverse parents’ decision-making about the human papillomavirus (HPV) vaccine: a codesign study
Source: BMJ Open. 2024 Sep 12;14(9):e079539. doi: 10.1136/bmjopen-2023-079539 (PMC11409246; doi:10.1136/bmjopen-2023-079539)
Supplement: online supplemental file 3 [file bmjopen-14-9-s003.pdf]

Purpose: Raise knowledge and understanding among parents about HPV, HPV-related diseases, HPV vaccine, and process of how the HPV vaccine was developed

Participant: NAME Paediatrician and Vaccine Scientist
Interviewed by parent: NAME

Activities: NAME and NAME to discuss the headline news stories:
HPV is making cervical cancer a 'thing of the past': Jabs prevents nearly 90% of cases | Daily Mail Online
https://www.bbc.co.uk/news/health-59148620
https://www.gov.uk/government/news/jcvi-advises-move-to-1-dose-of-hpv-vaccine-for-adolescents
https://www.bbc.co.uk/news/health-62438214
https://www.thelancet.com/journals/lancet/article/PIIS0140-6736(21)02178-4/fulltext

Question - BBC and mail online say HPV vaccine will make cervical caner a thing of the past - do you agree with this?

Question - BBC online stated that the JCVI advises that the UK should move to one dose of the HPV for teens. Why is that?

How can we trust this information?

Reliable opinion sources will tell you where they got their information, avoid exciting language, and make checking their sources relatively easy. Ideally, any information you accept should be available from at least two principal sources that you trust. If only one source is telling you something, be cautious.

You can spot 'fake news' by:
1. Developing a critical mindset.
2. Checking the source.
3. See who else is reporting the story.
4. Examining the evidence.
5. Not taking images at face value.
6. Checking that it "sounds right."

Key: Boxes in blue are key content to be covered in detail

Content to cover

**Table 1: HPV and HPV-related diseases**	
**What is HPV?**	Human papillomavirus, or HPV, is the name of a group of common viruses that live in thin, flat cells called 'epithelial cells'. These are found on body surfaces including the skin and internal linings of the mouth airways and vagina.<br><br>There are over 100 types of HPV. Each one is grouped as either 'high-risk' or 'low-risk'. Low-risk HPV types cause warts including genital warts and do not cause cancer. Some high-risk HPV types can cause serious illness including cancer.<br><br>HPV is responsible for almost all cases of genital warts and cervical cancer and many cases of other rarer cancers:<br><br>• 90% of anal cancers<br>• 78% of vaginal cancers<br>• 25% of vulvar (women's external genitals) cancers<br>• 50% of cancers affecting the penis

	• 60% of oropharyngeal (mouth and throat) cancers
**How can somebody get HPV?**	HPV can spread easily between people during any intimate sexual contact. As most people with HPV do not have any symptoms it is common for someone to infect another person without knowing about it.<br><br>Before the HPV vaccination programme was introduced, nearly everyone became infected with HPV within a few years of being sexually active.<br><br>HPV is very common and nothing to be ashamed about. It is not possible to know exactly how or when somebody is infected by HPV. Even couples in a long-term monogamous relationship can be affected because of exposures before they met.
**How does HPV cause cancer?**	When high-risk HPV infects cells, the virus interferes with the ways in which these cells communicate with one another. This can cause infected cells to multiply in an uncontrolled manner. Usually, the body's immune system recognises and destroys cells that are infected with HPV. The person does not know they have been infected with HPV or have any health problems.<br><br>However, in some cases the infected cells persist and continue to grow. Eventually, these precancerous cells can develop into cancer. Research has shown that infection with HPV usually begins many years before cancer is diagnosed.
How many people are affected by HPV-related cancers each year?<br><br>(MacMillan/Cancer Research)	Each year in the United Kingdom approximately 11,210 people are diagnosed with an HPV-related cancer.<br><br>This includes 4,260 women affected by cervical, vaginal and vulval cancers, 350 men diagnosed with penile cancer, 1350 men and women who develop anal cancer (more common in women than men), and 5,250 men and women diagnosed with oropharyngeal cancers (more common in men).

**Table 2. Vaccine development**	
**How does the HPV vaccine work?**	In the early 1990s, vaccine scientists at the University of Queensland, Australia, created proteins found on the outer layer of the HPV. The proteins were created in yeast cells in the lab. Because the proteins assemble to look like a viral particle, scientists refer to them as 'virus-like particles'.<br><br>Injecting these 'virus-like particles' as a vaccine, simulates the immune system to produce antibodies. In future encounters with HPV, these antibodies bind to the virus and prevent it from infecting cells. The HPV vaccine is better at inducing a protective immune response than infection with HPV itself.
**How do I know HPV vaccine produced in a safe way?**	A wish to understand the vaccine development process are natural. Just like all medical therapies, vaccines involve an initial experimental stage, which is then followed by further testing and validation for safety and efficacy. Scientists do not always do a good job at explaining these processes and their results in ways that are easy to understand. It is good to be curious about this process and ask questions to help understand it.<br><br>The HPV vaccine went through multiple rounds of testing in clinical trials with hundreds of thousands of participants in countries across the world.

	The UK Medical and Healthcare Products Regulatory Agency (MHRA) only approved the vaccines once they had reviewed all the clinical trial data. Now, over 100 countries have included the HPV vaccine within their immunisation schedule.<br><br>Even after the HPV vaccine was approved, the MHRA continues to monitor the safety and side-effects of the HPV vaccine. All side-effects are reported and tracked to help scientists and researchers understand more about the effectiveness and safety of the vaccines, and the best way to use them. If serious safety concerns are raised about any vaccine, its use may be stopped by MHRA. This has never happened for HPV vaccines.<br><br>Continuous monitoring means that there are available data from millions of people that give a complete picture of HPV vaccine safety and effectiveness. To date they have been shown to be highly effective and without any serious side effects<br><br>You can report any side effects that you think may be linked to the HPV vaccine using the Yellow Card hotline on 0808 100 3352 or report side effects online.
**What are ingredients of the vaccine?**	The latest version of the vaccine contains individual proteins from nine types of HPV virus, which produce a broad immune response. Apart from the active ingredients (the antigens), it also contains very small amounts of these other ingredients:<br><br>Aluminium, which strengthens the immune response to the vaccine<br>polysorbate 80, used as an emulsifier (to hold other ingredients together)<br>an amino acid called histidine, used as an acidity regulator<br>sodium chloride (ordinary table salt)<br>The vaccine DOES NOT contain genetic material from the HPV and therefore cannot cause an infection with HPV or HPV-related cancer.<br>The vaccine DOES NOT contain porcine or egg products.
**How effective is the HPV vaccine?**	The HPV vaccine is one of the most effective vaccines available. It provides almost 100% protection against infection from nine HPV types: including two low-risk HPV types that cause most genital warts and seven high-risk HPV types that cause most HPV-related cancers.<br><br>Studies around the world have found falling rates of precancerous changes in young people since the vaccine was introduced as well as reductions in cervical cancer cases.<br><br>Protection is expected to be long-lasting and is probably life-long. The latest research shows the vaccine still offers close to 100% protection more than 14 years after it was received, and this protection shows no sign of weakening. This research is ongoing.
**How can I get my adolescent child vaccinated?**	In England, young people aged 12 to 13 years are routinely offered the HPV vaccine when they are in Year 8 at secondary school.<br><br>If your adolescent child has missed the vaccination at school, they can have it up until their 25th birthday. Speak to your school immunisation team or GP surgery and make an appointment to have the missed dose as soon as possible.

**How many doses of the HPV vaccine will be adolescent child have?**	There is now enough evidence that just one vaccine dose provides excellent protection against cancers caused by a virus called by HPV.<br><br>The evidence comes from clinical trials which show that immunity from one dose of the HPV vaccine gives as good protection as from two doses and immunity is likely to last for at least 10 years. By changing from a two-dose schedule to a one-dose schedule, inconvenience and needle burden on young people will also be reduced.

Interview with consultants & survivors of HPV-related cancer

Purpose: Raise awareness and understanding of the potential consequences of an HPV infection

Participants: NAMES

Include: Filming of treatment rooms / equipment within HOSPITAL

**Table 1. Treatment for HPV and HPV-related diseases**	
**How would someone know they have HPV?**	Most people with HPV - no matter what their gender is - do not have any symptoms or even realise they have an infection.<br><br>Sometimes people with HPV can develop genital warts. The type of HPV that causes genital warts does not cause cancer.
**Which cancers are caused by HPV virus?**	Women can develop cervical, vaginal and vulval cancer<br>Men can develop penile cancer<br>Oro-pharyngeal (Head and neck) and anal cancer can occur in either sex
**What symptoms could I have from an HPV-related cancer?**	The symptoms are specific to each type of cancer.<br><br>In the case of cervical cancer, the most common symptom is irregular vaginal bleeding or bleeding after sex. Some women may notice changes to vaginal discharge. People with an HPV-related mouth and neck cancer may notice a painless swelling or lump in their neck.<br><br>More information about signs and symptoms of the different HPV-related cancers can be found on the NHS or Macmillan websites.
**Are there any tests available to detect HPV?**	HPV testing is routinely provided as part of the national NHS cervical screening programme. As well as checking for abnormal cell changes in the cervix, the sample is tested for the presence of high-risk HPV. It is not a test for cancer. Cells are only examined if HPV virus is detected.<br><br>Abnormal cells detected on the cervix at an early stage can be removed or destroyed through treatments. This usually prevents the abnormal cells from developing into cancer.<br><br>It is important to attend for cervical cancer screening even if you have had the HPV vaccination.
**Are there any tests that can detect cell changes for other HPV-related cancers?**	There are no tests or screening programme yet to detect for other HPV-related cancers available on the NHS. This is because the tests are not yet useful and result would not help the management or treatment of any other HPV-related cancer, apart from cervical cancer.

**What are the treatment options for HPV-related cancers?**	Treatment options include surgery, chemotherapy and radiation. The most suitable option will depend on the type of cancer, how big it is, whether it has spread to anywhere else in the body, and the patients' general health.<br><br>In most hospitals, a multi-disciplinary team of specialists will decide which treatment or combination of treatments will be best. Individual preferences will also be considered.
**What are treatment options for cervical cancer detected at an earlier stage?**	Cervical cancer detected at an early stage can often be treated with surgery. This includes a large loop excision of the transformation zone (a type of surgery that removes a small part of the cervix), and a cone biopsy (a type of surgery that removes a cone shaped part of the cervix).
**What are treatment options for cervical cancer detected at a later stage?**	For cervical cancer detected at a later stage, surgeries including a trachelectomy (removal of the cervix) or hysterectomy (removal of the womb or uterus) could be required.<br><br>Other types of treatment after surgery may also be offered to help treat the cervical cancer and reduce the risk of cervical cancer coming back. This will depend on factors such as the stage of cervical cancer and whether the patient has had any previous treatments.
Radiotherapy	Radiotherapy is a type of treatment that destroys cervical cancer cells using high energy x-rays. A machine is used to deliver radiotherapy from outside the body.
Chemotherapy	Chemotherapy uses drugs to destroy cancer cells. The drugs travel around the body in the bloodstream to help stop cancer cells from growing.<br><br>A single drug or a combination of drugs are given in cycles. The frequency of chemotherapy and number of cycles will depend on the cervical cancer stage and drugs you are given.<br><br>Radiotherapy and chemotherapy may be given together.
Brachytherapy	Brachytherapy treatment is internal radiography. This treatment may be started after chemotherapy, radiotherapy, or surgery.<br><br>Radioactive material is placed inside the patient's womb or vagina so it can destroy the cancer cells.
**What can be affected by cancers caused by HPV?**	Anyone. Men and women of different ages, backgrounds and ethnic groups are being treated for HPV-related cancers.
**What effects could I having following treatment for cancer?**	There are both short- and longer-term side effects following treatment for cancer. Symptoms may slowly get better over 1 or 2 years after treatment ends, or even longer. Sometimes long-term effects are permanent.
**Could having treatment for cancer affect my fertility?**	Cancer treatments can affect the fertility of both men and women. Some women may experience early menopause as a result of their treatment. Fertility specialists will be able to advise on options (e.g. egg and sperm storage) where patients wish to have a child in the future.

Table 2. Patient story
Diagnosis<br>How did you find out you had this cancer?<br>What was your first reaction when you found out?<br>Did you have any symptoms of having this type of cancer?<br>How did the diagnosis impact you emotionally?<br>How did the diagnosis impact your friends and/or family?<br><br>*Treatment*<br>Can you tell what about the treatment you had?<br>How long did it take?<br>How long did you stay in hospital for?<br>Were there any immediate side-effects from the treatment?<br>Did you have any longer term side-effects from the treatment?<br><br>How did the treatment impact you emotionally?<br>- What support did you have available to you?<br>How did the treatment impact you practically?<br>How did the treatment impact your day-to-day life?<br>Were you able to keep working?<br>How did the diagnosis impact your family?<br><br>*Future*<br>How do you feel now about everything you have been through?<br>What have been the most far reaching effects of having had this diagnosis?<br>What would you say to a parent who is deciding whether their adolescent child should have the HPV vaccine?

Interview with healthcare professional: GP & immunisation nurses (Bristol & London)
Purpose of filming shoot: Address concerns commonly raised by parents & provide reassurance
Participants: NAMES

Blue: Important to include

- *Take written consent from each participant*
- *Provide an overview of the HPV vaccination programme*
- *Explain the purpose of COMMUNICATE study to parents*
- *Explain the outline of the shoot*

1. **Workshop activity with parents (morning)**

- *Explain to parents that they are going to be filmed asking other parents what they think about the HPV vaccine.*
- *Ask parents to think about when they first heard about the HPV vaccine and to come up with questions / points they might discuss with other parents*
- *Ask parents if they think the questions / points listed below are important as well*
- *Ask parents to select with four questions that they would like to ask parents with vaccinated children*

TABLE 1. Guiding questions for morning shoot

I received a letter from the school today asking me to give consent for my son/daughter to have the HPV vaccine. What did you do when you received this letter?<br><br>The immunisation nurses rang me today to ask if I wanted my son/daughter to have the HPV vaccine. But I didn't feel comfortable giving consent over the phone because I hadn't heard about it before.<br><br>I never received any information about the HPV vaccine from school so my son/daughter hasn't had it. What do you think I should do?<br><br>Why did you decide to give your son/daughter the HPV vaccine?<br><br>I heard that by giving the HPV vaccine parents are giving the green light for their children to have lots of sexual partners. Do you think that is true?<br><br>I am teaching my son/daughter to keep safe by not having lots of sexual partners.<br><br>I don't think the vaccine is necessary because HPV is a sexually transmitted infection and my son/daughter won't have sex until they are married. What do you think about that?<br><br>Do you think it's safe for my son/daughter to have the HPV vaccine?<br><br>What side-effects did your son/daughter have after having the HPV vaccine?<br><br>How long did the side-effects last?<br><br>How was it for your son/daughter having the HPV vaccine at school?

2. Workshop activity with parents (afternoon)

- *Ask parents to consider the following question: '**What do you think the biggest concerns are that parents have about vaccinating their adolescent child against HPV?**'*
- Ask parents to indicate whether the following concerns listed in Table 2 are important concerns as well (if not mentioned).
- *If any of the concerns are the same as that parents in the workshop have come up with, group them together.*
- *Ask parents to prioritise the most important concerns to be addressed by a healthcare professional.*
- *Ask parents to select four of the concerns they want to ask the healthcare professional during the filming in the activity*
- *Ask for feedback on suggested responses listed (Tables 2-5) (optional)*

TABLE 2. Concerns parents have about the HPV vaccine

Safety and side effects<br>▪ **What are the side-effects of the HPV vaccine?**<br>▪ My friend's adolescent child fainted after having the HPV vaccine. Was that a side-effect?<br>▪ Does the HPV vaccine cause infertility?<br>▪ I would be worried about giving my adolescent child the HPV vaccine as I saw on social media that the HPV vaccine can cause cancer.<br>▪ My adolescent child has a long-term health condition. Can they have the vaccine?

- I have heard that the HPV vaccine can cause serious side-effects.
- How safe is it to give my adolescent child the HPV vaccine?

Vaccine beliefs

- I think too many vaccines offered to teenagers. I worry that it could overload their immune system.
- I would prefer to use more natural methods to support the immune system of my adolescent child.

Sexual activity

- If I give permission for my adolescent child to have the HPV vaccine, does this give the green light for my adolescent child to be sexually active?
- My adolescent child is not even close to being sexually active. I would rather wait until they are older to make the decision themselves.

Perceptions of need

- The HPV vaccine wasn't available when I was a child and I never got cervical cancer. Why does my son or daughter need the vaccine?
- Boys can't get cervical cancer. Why does my adolescent son need to have the HPV vaccine?

Reactance

- **I am tired of the government telling me that my son or daughter has to have another vaccine.**

**TABLE 2. SAFETY & SIDE EFFECTS**	
**What are the side-effects of the HPV vaccine?**	As with any medicine, there may be some side-effects after your adolescent child has had the HPV vaccine. These are usually mild and should only last a day or two. These could be a sore arm, pain and swelling where the injection is given. Other less common side-effects that affect up to 1 in 10 young people include vomiting, feeling hot and shivery (fever), or sickness (nausea).<br><br>These side-effects are normal signs that our bodies are building protection and that our immune systems are working. These side-effects should go away in a few days.<br><br>I know that it can be inconvenient if your adolescent child experiences side-effects, and I understand that as parents we would rather avoid the inconvenience. Mild side-effects are a minor inconvenience compared to the severe consequences your adolescent child could suffer from an HPV-related cancer after not having the vaccine.<br><br>Very rarely, about one in a million times, a severe allergic reaction (called anaphylaxis) may happen after the HPV vaccine is administered. This is roughly the same as being struck by lightning in a given year.

	This is usually because the person is allergic to an ingredient in the vaccine (e.g. yeast). This is similar to rates of allergic reactions to other vaccines given to children and adolescents.<br><br>A severe allergic reaction is treatable and usually happens shortly having the HPV vaccine. All health care professionals involved in giving vaccinations are fully trained to treat this.
**My friend's adolescent child fainted after having the HPV vaccine.**<br><br>**Was that a side-effect?**	It is not unusual for young people to feel faint before or after having the HPV vaccine. Often this is because young people feel nervous about having the injection, rather than being caused by the HPV vaccine itself.<br><br>As with any medicine, there may be some side-effects after your adolescent child has had the HPV vaccine. These are usually mild and should only last a day or two. These could be a sore arm, pain and swelling where the injection is given. Other less common side-effects that affect up to 1 in 10 young people include vomiting, feeling hot and shivery (fever), or sickness (nausea).
**Does the HPV vaccine cause infertility?**	It is normal to have questions and doubts about medical treatments and how they might affect us. It is understandable that we want to know what caused a problem so we can try to avoid it or solve it.<br><br>The HPV vaccine actually protects fertility. HPV infections can cause cervical cancer, which is a great risk to fertility. The HPV vaccine is effective at preventing cervical cancer so it can help decrease the number of women unable to have a baby.
**I would be worried about giving my adolescent child the HPV vaccine as I saw on social media that the HPV vaccine can cause cancer.**	It is normal to have questions and doubts about medical treatments and how they might affect us. It is understandable that we want to know what caused a problem so we can try to avoid it or solve it. It's also great that you are asking more about the information you have seen.<br><br>The HPV vaccine is made using only a single protein from the surface of the virus. As it is only part of the virus it cannot cause an infection with HPV, which could lead to the development of cancer.<br><br>Research from studies across the world has shown that the HPV vaccine is safe and there are no links having the HPV vaccine to developing cancer.
**My adolescent child has a long-term health condition. Can they have the vaccine?**	There are indeed specific situations where vaccinations are not recommended. It is therefore advisable that you ask questions if their child has a long-term condition that they worry about.<br><br>However, there are very few individuals who cannot receive HPV vaccine. For example, we wouldn't recommend the HPV vaccine if your adolescent child has had previous allergic reactions (or anaphylactic reaction) to ingredients within the HPV vaccine, like yeast.<br><br>Healthcare professionals will always confirm whether the HPV vaccine in suitable for someone with a long-term health condition.
**I have heard that the HPV vaccine can cause serious side-effects.**	It is normal to have questions and doubts about medical treatments and how they might affect us. Vaccines, like all medicines, do have side effects. With the HPV vaccine, these are usually mild and will only last day or two.

**How safe is it to give my adolescent child the HPV vaccine?**	Sometimes unrelated medical conditions occur right after the HPV vaccine is given to an adolescent child, but so do other accidents. It is important to distinguish between events that simply occur at random close together, and one that is caused by another. Not everything that happens after someone has a vaccine is *caused* by the vaccine—it may be entirely unrelated.<br><br>If your teenager falls over when texting on their phone after having the HPV vaccine, that would be inconvenient, but it would not be because of the vaccine. Only careful research can determine whether a vaccine is to blame for what happens to someone after a vaccination.

**TABLE 3. VACCINE NORMS**	
I think too many vaccines offered to teenagers.<br><br>I worry that it could overload their immune system.	It is reasonable to worry about how your adolescent child's body might react to a medical treatment. Some people may be concerned that vaccines are being overused because other medicines, like antibiotics, have sometimes been prescribed when they were not needed.<br><br>A review of scientific evidence by the World Health Organisation found no evidence that being vaccinated with multiple vaccines at any age can weaken or harm the immune system.<br><br>In fact, vaccines work by strengthening the immune system, training it to recognise viruses and to fight against them. Without vaccines, the immune system risks being overloaded by trying to fight off infections without training.
I would prefer to use more natural methods to support the immune system of my adolescent child	Infection with HPV can be difficult to avoid as everyone who is ever sexually active is at risk. Sometimes people can be infected with HPV and not know it. Condoms are not completely effective in preventing HPV infections.<br><br>The HPV vaccine is another important part of a healthy lifestyle, because it trains our body to fight off an infection with HPV without needing to expose ourselves to the full risk of developing an HPV-related cancer. Research has shown the HPV vaccine is better at inducing an immune response than the infection with HPV itself.<br><br>Having your adolescent vaccinated against HPV, as well as providing them with education at home or school about keeping safe, will help keep your adolescent child healthy.

**TABLE 4. SEXUAL ACTIVITY**	
If I give permission for my adolescent child to have the HPV vaccine, does this give the green light for my child to be sexually promiscuous?	It is normal to be concerned about your adolescent child's health, including their sexual health. Some parents are understandably concerned that if their adolescent child has the HPV vaccine, this will encourage sexual activity at a young age.<br><br>As HPV is usually transmitted through sexual contact, it is important that adolescent children are vaccinated before they become sexually active. This is why the vaccine is offered to young people aged 12 to 13 years old.<br><br>You are not endorsing or promoting sexual activity if you decide to vaccinate your child. There is no evidence that young people who receive

	the vaccine start sexual relations earlier or have more sexual partners. Rather, the HPV vaccine protects your adolescent child for when they do become sexually active, which may be many years after they receive the vaccine.
My adolescent child is not even close to being sexually active.<br><br>I would rather wait until they are older to make the decision themselves.	It's normal to feel that you would prefer to wait with vaccination until your adolescent child needs it because they have intimate relationships. This stage of life may indeed be a long way off for your adolescent child.<br><br>The vaccine works best if it is given before exposure to HPV – that is, before sexual activity begins. We also know that younger people create more antibodies when given the vaccine than older teenagers do. This means they are better protected if they are exposed to HPV in the future.<br><br>The purpose of the HPV vaccine is the same as any other vaccine – it is to protect your adolescent child not just in the immediate future, but over the course of their life.<br><br>If your adolescent child has the vaccine at the recommend age, you can feel confident that you have done your best to protect them from HPV and some of the diseases it can cause when they are older and thinking about starting intimate relationships.

**TABLE 5. PERCEPTIONS OF NEED**	
**The HPV vaccine wasn't available when I was a child and I never got cervical cancer.**<br><br>**Why does my son or daughter need the vaccine?**	Since most people are not aware if they have had an HPV infection, or do not know anyone who has developed an HPV-related cancer, it is normal to wonder if your adolescent children would be affected by HPV.<br><br>HPV is very common, and indeed in many cases it does not cause any problems. But it is impossible to know in advance whether your adolescent child would be severely affected and develop cancer.<br><br>Evidence from clinical trials showed that showed that the HPV vaccination programme would reduce the number and deaths from HPV-related cancers, as well as a significant number of cervical screening abnormalities.<br><br>That is was the government decided to introduce the HPV vaccination programme in the UK after extensive consultation with scientists, epidemiologists and public health experts.
**Boys can't get cervical cancer. Why does my adolescent son need to have the HPV vaccine?**	It's true that a lot of the coverage about HPV has been on cervical cancer, as it is the most common severe consequence. HPV can also cause cancers of the head and neck, or penile and anal cancers that affect men. In fact, about 4 of every 10 cases of HPV-related cancers occur in boys or men<br><br>This is why from 2019 young men have been included in the HPV vaccination programme. HPV is a known cause of cervical cancer. It can also cause.<br><br>Because vaccinating boys will also decrease the spread of the virus, they will not only protect themselves, but also their sexual partners.

**TABLE 6. REACTANCE**

**I am tired of the government telling me that my son or daughter has to have another vaccine**	Everyone should indeed have the autonomy to make decisions about their medical care. It is appropriate to fight back against condescending or coercive messages. Everyone has the right to make decisions that are not dictated by others.<br><br>But when we have autonomy over our medical decisions, we are also responsible for the consequences of our decision—such as falling ill or making others ill. We thus need to think carefully about what those consequences will be, both for ourselves and for others around us, like our families and friends.<br><br>The HPV vaccine has the power to protect young people from potentially life-threatening diseases - cancer. Making the decision about the HPV vaccine could be one of the most empowering medical decisions you make with your adolescent child.
