# Supplementary material for: Developing films to support vaccine-hesitant, ethnically diverse parents’ decision-making about the human papillomavirus (HPV) vaccine: a codesign study
Source: BMJ Open. 2024 Sep 12;14(9):e079539. doi: 10.1136/bmjopen-2023-079539 (PMC11409246; doi:10.1136/bmjopen-2023-079539)
Supplement: online supplemental file 4 [file bmjopen-14-9-s004.pdf]

<u>Topic Guide</u>

Explain purpose of project and session.

- We are part of a team working on the COMMUNICATE study which aims to co-produce a series of films to support parents' decision making and improve uptake of the HPV vaccination programme.
- For the study we have developed a series of films in collaboration with the creative team at Hide The Shark, parents and key stakeholders
- We are now seeking feedback from parents and key stakeholders to ensure that the films are fit for purpose
- We anticipate that the session will last up to two hours (workshop) or 45 minutes (interview)

Aim

- Today, we would like to ask for your feedback on the films we have developed as part of the COMMUNICATE study
- Explain the conversation is being recorded so the researcher present can make notes after.
- Answer any questions they may have.
- Ensure either written or verbal consent is obtained from the participant.

The COMMUNICATE films

The films are intended to be provided to parents to help support decision-making about the HPV vaccine for their adolescent child. They may be provided before the young person is offered the HPV vaccine at their school or at a later stage. It aims to help parents make more informed decisions by equipping them with knowledge about the HPV vaccination programme and providing reassurance for their adolescent child to be vaccinated.

- Overall, what do you think about using the films in this way?
- What are your reasons for this?

Ask the participant to provide a commentary on the following components of the package.

- *Film 1. XX*
- *Film 2. XX*
- *Film 3. XX*

Question prompts: Films

- Overall, what do you think about the film about X?
- What do you like about the film?
- Is there anything you do not like about the film?

- What message do you think the film was trying to communicate?
- How relevant do you think the messages of the film are to parents?
- Can you think of any other messages or content that could be included?
- Is there any messaging or content that you think could be left out?

What do you think of the style?

Tone - humour / serious?

Representation of people? (gender, ethnicity, sexuality, settings)

What do you think about the font or typology?
Bigger? Smaller?
Does the colour stand out enough?
Is it easy to read?

Overall
What did you think of the films?
Are there any improvements we could make?
Are there any additional content or messages that you think are important to include?

What did you think about the order the content was presented?
Are there any other routes to sign post parents that should be included?
Are you happy for your organisation’s logo to be included? [IF APPLICABLE]

Finally, is there anything else you would like to tell me or ask me about?
Many thanks for taking part in this interview.

[NOTE: *ensure that the participant is given the £30 gift voucher*]
