# Supplementary material for: Developing films to support vaccine-hesitant, ethnically diverse parents’ decision-making about the human papillomavirus (HPV) vaccine: a codesign study
Source: BMJ Open. 2024 Sep 12;14(9):e079539. doi: 10.1136/bmjopen-2023-079539 (PMC11409246; doi:10.1136/bmjopen-2023-079539)
Supplement: online supplemental file 5 [file bmjopen-14-9-s005.pdf]

Supplementary File 5.. Evidence for key behavioural determinants that the COMMUNICATE films are trying to address

BEHAVIOURAL ISSUE	BEHAVIOURAL DETERMINANTS	EVIDENCE FOR BEHAVIOUR	KEY FINDING
Lower HPV vaccination uptake among young people from minority ethnic groups & more deprived backgrounds	1. Low levels of understanding about the HPV vaccine among some parents	Hendry, M., et al., “HPV? Never heard of it!”: A systematic review of girls’ and parents’ information needs, views and preferences about human papillomavirus vaccination. Vaccine, 2013. 31(45): p. 5152-5167. [1]	- Parents had insufficient knowledge and understanding about the HPV vaccine.<br>- Could impact on ability to make informed choices.
		Lacombe-Duncan, A., P.A. Newman, and P. Baiden, Human papillomavirus vaccine acceptability and decision-making among adolescent boys and parents: A meta-ethnography of qualitative studies. Vaccine, 2018. 36(19): p. 2545-2558. [2]	- Lack of awareness about HPV vaccines and benefits for boys influenced vaccine acceptability.
		Marshall, S., et al., Views of parents regarding human papillomavirus vaccination: A systematic review and meta-ethnographic synthesis of qualitative literature. Research in Social and Administrative Pharmacy, 2019. 15(4): p. 331-337. [3]	- Limited parental knowledge of HPV, even in those who have already made vaccination decisions for their daughter.
		Netfa, F., et al., Knowledge, Attitudes and Perceptions of Immigrant Parents Towards Human Papillomavirus (HPV) Vaccination: A Systematic Review. Tropical Medicine and Infectious Disease, 2020. 5(2): p. 58. [4]	- Gaps in knowledge including the HPV vaccine and its relation to cancer among immigrant parents in higher-income countries.
		Marlow, L.A.V., et al., Ethnic differences in human papillomavirus awareness and vaccine acceptability. Journal of Epidemiology and Community Health, 2009. 63(12): p. 1010-1015. [5]	- Compared to White British parents, there was lower awareness and acceptability of the HPV vaccine among ethnic minority women, particularly Indian, African and Bangladeshi women.
		Gordon, D., J. Waller, and L.A. Marlow, Attitudes to HPV vaccination among mothers in the British Jewish community: reasons for accepting or declining the vaccine. Vaccine, 2011. **29**(43): p. 7350-6. [6]	- HPV and cervical cancer knowledge varied, with poor knowledge attributed to lack of contact with the disease.
		Batista Ferrer, H., et al., Barriers and facilitators to uptake of the school-based HPV vaccination programme in an ethnically diverse group of young women. Journal of Public Health, 2016. 38(3): p. 569-577. [7]	- Evidence for unmet information needs due to language and literacy barriers, with important implications for informed consent.
		Mupandawana, E.T. and R. Cross, Attitudes towards human papillomavirus vaccination among African parents in a city in the north of England: a qualitative study. Reproductive health, 2016. **13**(1): p. 1-12. [8]	- HPV and cervical cancer awareness was generally low, with awareness lower in fathers.<br>- Information about why the vaccine is necessary was generally misunderstood.

		Creed, S., E. Walsh, and T. Foley, A qualitative study of parental views of HPV vaccination in Ireland. European Journal of General Practice, 2021. 27(1): p. 1-9. [9]	- Gaps in knowledge included the under-estimation of the prevalence of HPV infection and the specific reasons for vaccination timing.
		Fisher, H., et al., Information needs of ethnically diverse, vaccine-hesitant parents during decision-making about the HPV vaccine for their adolescent child: A qualitative study. Under review [10]	- Underpinning the decision-making process among many of the parents interviewed was limited knowledge and misunderstandings about HPV and the HPV vaccine.
	2. Perceptions of protection offered	Hendry, M., et al., "HPV? Never heard of it!": A systematic review of girls' and parents' information needs, views and preferences about human papillomavirus vaccination. Vaccine, 2013. 31(45): p. 5152-5167. [1]	- Parents were motivated to vaccinate their daughters by the clear benefit of cervical cancer prevention.
		Batista Ferrer, H., et al., Barriers and facilitators to HPV vaccination of young women in high-income countries: a qualitative systematic review and evidence synthesis. BMC Public Health, 2014. 14(1): p. 700. [11]	- Parental decision-making in favour of the vaccine was motivated by protection against HPV acquisition or the development of HPV-related conditions
		Lacombe-Duncan, A., P.A. Newman, and P. Baiden, Human papillomavirus vaccine acceptability and decision-making among adolescent boys and parents: A meta-ethnography of qualitative studies. Vaccine, 2018. 36(19): p. 2545-2558. [2]	- Perceptions of protecting their sons against HPV-related cancers and genital warts improved acceptability of the HPV vaccine.
		Marshall, S., et al., Views of parents regarding human papillomavirus vaccination: A systematic review and meta-ethnographic synthesis of qualitative literature. Research in Social and Administrative Pharmacy, 2019. 15(4): p. 331-337. [3]	- Fear of harm from HPV-related cancers motivated parents to vaccinate their children.
		Gordon, D., J. Waller, and L.A. Marlow, Attitudes to HPV vaccination among mothers in the British Jewish community: reasons for accepting or declining the vaccine. Vaccine, 2011. **29**(43): p. 7350-6. [6]	- Mothers who accepted the vaccine generally did so to protect their daughters health.
		Mupandawana, E.T. and R. Cross, Attitudes towards human papillomavirus vaccination among African parents in a city in the north of England: a qualitative study. Reproductive health, 2016. **13**(1): p. 1-12. [8]	- HPV and cervical cancer perceived as a white person's disease.
		Creed, S., E. Walsh, and T. Foley, A qualitative study of parental views of HPV vaccination in Ireland. European Journal of General Practice, 2021. 27(1): p. 1-9. [9]	- Desire to protect their daughter and prevent disease.
		Fisher, H., et al., Information needs of ethnically diverse, vaccine-hesitant parents during decision-making about the HPV vaccine for their adolescent child: A qualitative study, Under review [10]	- Often parents appeared to be uncertain about the balance of risk and benefit of the vaccine. Content of future communication materials should emphasise the clinical sequelae of HPV, protection offered against HPV-related diseases, universality of health benefits by

			gender, and the effectiveness of the HPV vaccine and vaccination programme.
	3. Sexual transmission of HPV	Hendry, M., et al., “HPV? Never heard of it!”: A systematic review of girls’ and parents’ information needs, views and preferences about human papillomavirus vaccination. Vaccine, 2013. 31(45): p. 5152-5167. [1]	- HPV vaccine could encourage young women to engage in sexual activity early or have multiple sexual partners.
		Batista Ferrer, H., et al., Barriers and facilitators to HPV vaccination of young women in high-income countries: a qualitative systematic review and evidence synthesis. BMC Public Health, 2014. 14(1): p. 700. [11]	- Parental perceptions of adolescent sexual behaviour influenced decision-making about the HPV vaccine.<br>- Parents with perceptions of low risk of HPV acquisition may delay vaccination of their daughter.
		Lacombe-Duncan, A., P.A. Newman, and P. Baiden, Human papillomavirus vaccine acceptability and decision-making among adolescent boys and parents: A meta-ethnography of qualitative studies. Vaccine, 2018. 36(19): p. 2545-2558. [2]	- Parents expressed apprehension about vaccination due to religious or cultural beliefs prohibiting sexual contact outside of marriage.<br>- Parents who believed their sons to be sexually inactive were less supportive of the HPV vaccine.
		Marshall, S., et al., Views of parents regarding human papillomavirus vaccination: A systematic review and meta-ethnographic synthesis of qualitative literature. Research in Social and Administrative Pharmacy, 2019. 15(4): p. 331-337. [3]	- Parents did not believe their adolescent children to be sexually active at the recommended aged of vaccination, especially relevant among population groups with religious beliefs.<br>- Parents were often concerned about the potential encouragement of sexual activity associated with vaccination.
		Netfa, F., et al., Knowledge, Attitudes and Perceptions of Immigrant Parents Towards Human Papillomavirus (HPV) Vaccination: A Systematic Review. Tropical Medicine and Infectious Disease, 2020. 5(2): p. 58. [4]	- Non-vaccinating ethnic minority parents believed HPV vaccination would encourage unsafe sexual practices and promiscuity.<br>- Reasons for declining included a belief that abstinence from sex before marriage would provide protection from disease.
		Marlow, L., J. Wardle, and J. Waller, Attitudes to HPV vaccination among ethnic minority mothers in the UK: An exploratory qualitative study. Human Vaccines, 2009. 5(2): p. 105-110. [6]	- Religious beliefs meant that mothers thought the vaccine would be less acceptable to other family members or would be perceived as unnecessary because of their low risk of HPV.
		Gordon, D., J. Waller, and L.A. Marlow, Attitudes to HPV vaccination among mothers in the British Jewish community: reasons for accepting or declining the vaccine. Vaccine, 2011. **29**(43): p. 7350-6. [6]	- Not perceived as necessary for their daughter, citing Jewish religious laws governing family purity and abstinence until marriage as reasons for daughter's low susceptibility.

		Batista Ferrer, H., et al., Barriers and facilitators to uptake of the school-based HPV vaccination programme in an ethnically diverse group of young women. Journal of Public Health, 2016. 38(3): p. 569-577. [7]	- Cultural values among families prohibiting sexual contact outside of marriage prevented young women from minority ethnic groups receiving the HPV vaccine.
		Mupandawana, E.T. and R. Cross, Attitudes towards human papillomavirus vaccination among African parents in a city in the north of England: a qualitative study. Reproductive health, 2016. **13**(1): p. 1-12. [8]	- Religion and good cultural upbringing contributed to in low-risk perceptions for their adolescent daughters.
		Fisher, H., et al., Information needs of ethnically diverse, vaccine-hesitant parents during decision-making about the HPV vaccine for their adolescent child: A qualitative study, Under review [10]	Stigma associated with the sexual transmissibility of HPV did not always negatively impact decision-making. However, some parents chose not to vaccinate on the basis of perceptions of low risk and a preference to provide education about sexual behaviours to their adolescent child.<br><br>Future communication materials, and related HPV messages, should directly address the misconceptions that parents voiced around being able to keep their adolescent children safe from the potential effects of HPV by educating them not to have sexual relations outside of marriage.
	Safety and side-effects	Hendry, M., et al., “HPV? Never heard of it!”: A systematic review of girls’ and parents’ information needs, views and preferences about human papillomavirus vaccination. Vaccine, 2013. 31(45): p. 5152-5167. [1]	- Potential for harm from adverse effects prevent vaccination of their daughters.
		Batista Ferrer, H., et al., Barriers and facilitators to HPV vaccination of young women in high-income countries: a qualitative systematic review and evidence synthesis. BMC Public Health, 2014. 14(1): p. 700. [11]	- Parents expressed worries concerning side-effects and safety of the HPV vaccine.
		Lacombe-Duncan, A., P.A. Newman, and P. Baiden, Human papillomavirus vaccine acceptability and decision-making among adolescent boys and parents: A meta-ethnography of qualitative studies. Vaccine, 2018. 36(19): p. 2545-2558. [2]	- Parents raised concerned with HPV vaccine safety.
		Marshall, S., et al., Views of parents regarding human papillomavirus vaccination: A systematic review and meta-ethnographic synthesis of qualitative literature. Research in Social and Administrative Pharmacy, 2019. 15(4): p. 331-337. [3]	- Safety and efficacy concerns, including the potential for impaired fertility, were raised by parents.

		Netfa, F., et al., Knowledge, Attitudes and Perceptions of Immigrant Parents Towards Human Papillomavirus (HPV) Vaccination: A Systematic Review. Tropical Medicine and Infectious Disease, 2020. 5(2): p. 58. [4]	- Non-vaccinating and partially vaccinating parents were concerned about potential side-effects.
		Marlow, L.A.V., et al., Ethnic differences in human papillomavirus awareness and vaccine acceptability. Journal of Epidemiology and Community Health, 2009. 63(12): p. 1010-1015. [5]	- Concerns about side-effects was a commonly raised barriers to vaccination regardless of ethnicity.
		Marlow, L.A.V., J. Wardle, and J. Waller, Attitudes to HPV vaccination among ethnic minority mothers in the UK: An exploratory qualitative study. Human Vaccines, 2009. 5(2): p. 105-110. [12]	- Potential for side-effects and the research processes for developing the vaccine were raised by non-vaccinating and partially vaccinating parents from various ethnic backgrounds.
		Gordon, D., J. Waller, and L.A. Marlow, Attitudes to HPV vaccination among mothers in the British Jewish community: reasons for accepting or declining the vaccine. Vaccine, 2011. **29**(43): p. 7350-6. [6]	- Concerns about the novelty of the vaccination resulted in declining the vaccine.
		Mupandawana, E.T. and R. Cross, Attitudes towards human papillomavirus vaccination among African parents in a city in the north of England: a qualitative study. Reproductive health, 2016. **13**(1): p. 1-12. [8]	- Concerns around unknown side effects, impact on fertility and novelty of the HPV vaccine were raised.
		Creed, S., E. Walsh, and T. Foley, A qualitative study of parental views of HPV vaccination in Ireland. European Journal of General Practice, 2021. 27(1): p. 1-9. [3]	- Fear of inflicting harm was a significant concern.
		Fisher, H., et al., Information needs of ethnically diverse, vaccine-hesitant parents during decision-making about the HPV vaccine for their adolescent child: A qualitative study, Under review [10]	- Misunderstanding around the potential of developing serious side-effects (e.g. fertility issues, developing cancer) were factors that could negatively impact decision-making by parents.
	4. Lack of engagement with consent procedures	Fisher, H., et al., Secondary analyses to test the impact on inequalities and uptake of the schools-based human papillomavirus (HPV) vaccination programme by stage of implementation of a new consent policy in the south-west of England. BMJ open, 2021. 11(7): p. e044980. [13]	- Lower return of consent form among families from more deprived backgrounds or ethnic minority groups
		Batista Ferrer, H., et al., Barriers and facilitators to HPV vaccination of young women in high-income countries: a qualitative systematic review and evidence synthesis. BMC Public Health, 2014. 14(1): p. 700 [11]	- Gaining consent in school-based programmes, where the parent is unlikely to attend during the vaccination procedure, act as a barrier to uptake.
		Batista Ferrer, H., et al., Barriers and facilitators to uptake of the school-based HPV vaccination programme in an ethnically diverse group of young women. Journal of Public Health, 2016. 38(3): p. 569-577. [7]	- A key issue that prevents uptake is non-returned parental consent forms.

		Audrey, S., et al., How acceptable is adolescent self-consent for the HPV vaccination: qualitative findings from a process evaluation in south-west England. Vaccine, 2020. 38(47): p. 7472-7478. [14]	- There was a strong presumption that parental consent should be sought, over allowing young people to provide themselves.
		Fisher H, Evans K, Ferrie J, Yates J, Roderick M & Audrey S. Young women's autonomy and information needs in the schools-based HPV vaccination programme: A qualitative study. *BMC Public Health* 2020; https://doi.org/10.1186/s12889-020-09815-x [15]	- Perceptions of adults as the decision-makers and targets for information, undermined opportunities for young women to be fully informed about the HPV vaccine and be involved in decisions affecting their health
		Paterson, P., Mounier-Jack, S., Saliba, V., Yarwood, J., White, J., Ramsay, M., & Chantler, T. Strengthening HPV vaccination delivery: findings from a qualitative service evaluation of the adolescent girls' HPV vaccination programme in England. *Journal of Public Health*. 2019 [16]	- Non-returned consent forms may either not have been given to parents, or were not completed by parents due to lack of time, misplacement, or hesitancy about HPV vaccination.
		Chantler, T., Letley, L., Paterson, P., Yarwood, J., Saliba, V., & Mounier-Jack, S. (2019). Optimising informed consent in school-based adolescent vaccination programmes in England: a multiple methods analysis. *Vaccine*, *37*(36), 5218-5224. [17]	- The non-return of consent forms was challenging to manage and of concern to immunisation teams since it could mean that adolescents were not receiving vaccines with significant health benefits.
		Chantler, T., et al., Does electronic consent improve the logistics and uptake of HPV vaccination in adolescent girls? A mixed-methods theory informed evaluation of a pilot intervention. BMJ Open, 2020. 10(11): p. e038963. [18]	- Language barriers accounted for some difficulties, but practical issues also played a role, for example, some parents had not signed up for the school parent mail system hence did not receive the weblink.

References

1. Hendry, M., et al., “HPV? Never heard of it!”: A systematic review of girls’ and parents’ information needs, views and preferences about human papillomavirus vaccination. Vaccine, 2013. **31**(45): p. 5152-5167.

2. Lacombe-Duncan, A., P.A. Newman, and P. Baiden, Human papillomavirus vaccine acceptability and decision-making among adolescent boys and parents: A meta-ethnography of qualitative studies. Vaccine, 2018. **36**(19): p. 2545-2558.

3. Marshall, S., et al., Views of parents regarding human papillomavirus vaccination: A systematic review and meta-ethnographic synthesis of qualitative literature. Research in Social and Administrative Pharmacy, 2019. **15**(4): p. 331-337.

4. Netfa, F., et al., Knowledge, Attitudes and Perceptions of Immigrant Parents Towards Human Papillomavirus (HPV) Vaccination: A Systematic Review. Tropical Medicine and Infectious Disease, 2020. **5**(2): p. 58.

5. Marlow, L.A.V., et al., Ethnic differences in human papillomavirus awareness and vaccine acceptability. Journal of Epidemiology and Community Health, 2009. **63**(12): p. 1010-1015.

6. Gordon, D., J. Waller, and L.A. Marlow, Attitudes to HPV vaccination among mothers in the British Jewish community: reasons for accepting or declining the vaccine. Vaccine, 2011. **29**(43): p. 7350-6.

7. Batista Ferrer, H., et al., Barriers and facilitators to uptake of the school-based HPV vaccination programme in an ethnically diverse group of young women. Journal of Public Health, 2016. **38**(3): p. 569-577.

8. Mupandawana, E.T. and R. Cross, Attitudes towards human papillomavirus vaccination among African parents in a city in the north of England: a qualitative study. Reproductive health, 2016. **13**(1): p. 1-12.

9. Creed, S., E. Walsh, and T. Foley, A qualitative study of parental views of HPV vaccination in Ireland. European Journal of General Practice, 2021. **27**(1): p. 1-9.

10. Fisher H, D.S., Audrey S, Finn A, Hajinur H, Mohamed A, Hickman M, Mounier-Jack S, Roderick M, Tucker L, Yates, J, & Chantler T., Information needs of ethnically diverse, vaccine-hesitant parents during decision-making about the. HPV vaccine for their adolescent child: A qualitative study Under review, 2023.

11. Ferrer, H.B., et al., Barriers and facilitators to HPV vaccination of young women in high-income countries: a qualitative systematic review and evidence synthesis. BMC Public Health, 2014. **14**(1): p. 700.

12. Marlow, L.A.V., J. Wardle, and J. Waller, Attitudes to HPV vaccination among ethnic minority mothers in the UK: An exploratory qualitative study. Human Vaccines, 2009. **5**(2): p. 105-110.

13. Fisher, H., et al., Secondary analyses to test the impact on inequalities and uptake of the schools-based human papillomavirus (HPV) vaccination programme by stage of implementation of a new consent policy in the south-west of England. BMJ open, 2021. **11**(7): p. e044980.

14. Audrey, S., et al., How acceptable is adolescent self-consent for the HPV vaccination: qualitative findings from a process evaluation in south-west England. Vaccine, 2020. **38**(47): p. 7472-7478.

15. Fisher, H., et al., Young women’s autonomy and information needs in the schools-based HPV vaccination programme: A qualitative study. BMC Public Health, 2020. **20**(1680).

16. Paterson, P., et al., Strengthening HPV vaccination delivery: findings from a qualitative service evaluation of the adolescent girls’ HPV vaccination programme in England. Journal of Public Health, 2019. **43**(1): p. 189-196.

17. Chantler, T., et al., Optimising informed consent in school-based adolescent vaccination programmes in England: A multiple methods analysis. Vaccine, 2019. **37**(36): p. 5218-5224.

18. Chantler, T., et al., Does electronic consent improve the logistics and uptake of HPV vaccination in adolescent girls? A mixed-methods theory informed evaluation of a pilot intervention. BMJ Open, 2020. **10**(11): p. e038963.
