# Supplementary material for: Developing films to support vaccine-hesitant, ethnically diverse parents’ decision-making about the human papillomavirus (HPV) vaccine: a codesign study
Source: BMJ Open. 2024 Sep 12;14(9):e079539. doi: 10.1136/bmjopen-2023-079539 (PMC11409246; doi:10.1136/bmjopen-2023-079539)
Supplement: online supplemental file 6 [file bmjopen-14-9-s006.pdf]

Supplementary File 6. Summary of findings from interviews with vaccine hesitant parents

THEMES & SUB-THEMES	KEY FINDINGS	EXEMPLAR QUOTE
(i) Mechanisms to raise the profile of the HPV vaccination programme		
**Credible sources of information**	Healthcare professionals with the relevant specialist knowledge were considered to be the most trusted source of information about the HPV vaccine. General practitioners and nurses were frequently referred to, as well as consultants involved in the treatment of HPV-related disease.<br><br>A few parents suggested vaccine scientists or immunologists could provide information about the development of the vaccine.	'*I think should be a healthcare professional. The vaccine is actually medical [intervention] because it goes into your body and if you react to that, who's gonna treat it? The doctors and nurses.*' [Participant 12, mother of unvaccinated teenage boys, Somali]<br><br>'*The scientist or people who actually made the actual vaccination just explaining to people how it works and what it's actually going to do to your body. For me, it'll help*.' [Participant 15, parent of unvaccinated teenage girl, Somali]
	Many parents discussed (sense checked) the HPV vaccine within their social networks. A few parents felt reassured that the HPV vaccine did not present risk in terms of side-effects because they had spoken to friends/family whose teenagers had already been vaccinated without issue.	'*I spoke to people – I remember my sister, her daughter had it a few years ago and then my neighbour, her son had it last year. I had a few people and they said, 'Oh, no. None of my kids had side effects.' I thought, 'Okay, that's fine*.' [Participant 14, mother of vaccinated teenage boy, British African]
	Some parents discussed the importance of engaging with faith leaders (e.g. pastors, Iman) to raise the profile of the HPV vaccine. However, more often parents considered that the information should be delivered by healthcare professionals.	'*I think definitely for the Muslim community or any community who are religious, I think maybe they need to have a conversation with a religious scholar. Someone that has a medical background and at the same time who are sensitive to their culture who then can tell them whether this is appropriate or not*.' [Participant 11, mother of unvaccinated teenage boy, Somali]<br><br>'*I think healthcare professionals people take more serious [than faith leaders]. I know that Asian people, they always listen to the doctor*.' [Participant 13, mother of unvaccinated teenage boy, Bengali]
	Online sources were also cited as an important way to verify, or gather information, about the HPV vaccine. These include the NHS website, their social media accounts and YouTube. Some parents indicated information online did not always support positive decision-making.	'*Google: 'What is the HPV vaccine?' Then follow different links to different things, I wouldn't take the first answer, that's definite*. [Participant 5, mother of unvaccinated teenager girl, White British]<br><br>'*There is so much fear mongering information online and in the public and before you know it you are saying no, no, no, I don't want to know*.' [Participant 20, mother of unvaccinated teenager girl and boy, Somali]

**Mechanisms to improve awareness**	Information about the HPV vaccination programme is routinely provided in English language by email or letter from the school on behalf of the immunisation team ahead of the scheduled vaccination session.<br><br>There was variation in the extent to which this appeared to address parents' information needs. Some parents felt the information was sufficient to inform their decision-making. However, other parents felt the information was insufficient and reported undertaking their own research to address their information needs.<br><br>Despite having vaccine-eligible adolescent children, some parents reported not receiving any information about the HPV vaccination programme.	'*My eldest had a letter from school offering the vaccine… It just explained how it stops, preventing cancer and it's recommended really*.' [Participant 18, mother of vaccinated teenage girls, Somali]<br><br>'*I remember having to look it up [information about the HPV vaccine]. There wasn't any information in it which I read which gave me any idea of the data*.' [Participant 2, father of unvaccinated teenage boys and girl, British Asian]<br><br>'*I wasn't given any information or I was never told about the HPV vaccination process by the school*.' [Participant 7, father of unvaccinated teenage girl, British South Asian]
	Language barriers also prevented some parents being able to understand the information received.	'*Loads of people, they have a language barrier and they don't read, but watching it, they can put subscription like these people nowadays, where they do Google translator*.' [Participant 12, mother of unvaccinated teenage boys, Somali]
	Almost all parents were supportive of videos to share information about the HPV vaccine, but would need to be easily accessible to ensure engagement.<br><br>Other ways of raising awareness of the HPV vaccination programme included: (i) text messages from General Practice or schools; (ii) face-to-face interactions at community health events; (iii) social media campaigns; (iv) providing information in the Red book.<br><br>Parents also expressed a need for information about the HPV vaccine to be shared by schools ahead of consent being sought.	
**(ii) Structure for videos**		
**Balancing risk and benefits**	Many parents expressed the need for balanced information on the pros and cons of the HPV vaccine to inform decision-making.	'*There shouldn't be any hidden truth if there is also a side effect about it [HPV vaccine] and how it could react negatively, people should know so they would make the choice whether to go with it or not*.' [Participant 6, father of unvaccinated teenage boy, Black Caribbean]

	Some parents suggested healthcare professionals withheld important information in relation to side-effects to ensure high uptake of the vaccination programme.	'*What I don't like is when health professionals pretend these things don't exist and make the vaccine the best thing since sliced bread because sometimes it can affect people negatively and ignoring that just makes it sound really more suspicious for parents because you think then, why should I trust them if they can't even admit there might be a potential mistake somewhere or somebody's body might not agree to it*.' [Participant 11, mother of unvaccinated teenage boy, Somali]
**Presenting risks and benefits**	A few parents suggested presenting the statistics (e.g. death rates, side-effects, allergic reactions, effectiveness of vaccination programme) through infographics as an acceptable way of proving this information.	'*What are the differences between statistics [incidence of HPV-related cancers] back before the HPV vaccine and statistics now? What's the difference between if you do have it and if you don't?*' [Participant 5, mother of unvaccinated teenage girl, White British]
**Representation**	Some parents discussed the importance of representation from their own, or different, communities.	'*I think if you're trying to create such kind of content, it should tend to carry all types of tribe and ethnicity …and also they will tend to know that it's not just for certain kinds of people*.' [Participant 6, father of unvaccinated teenage boy, Black Caribbean]
**Scenarios for film shoots**	There was strong agreement from parents that a personal testimony from a person who has experienced HPV-related disease would convey the importance of why their child should be vaccinated.	'*I think the personal testimony would be useful to sort of put a human face to it, 'cause a number isn't terribly relatable, but a face is*.' [Participant 1, mother of unvaccinated teenage boy, White British]
	Some parents felt it would be helpful to include parents discussing their choice about to have the adolescent child vaccinated and whether their adolescent child had experienced side effects or not.<br><br>A few parents did not value this scenario, indicating they would prefer information directly from healthcare professionals.	'*Having some parents talk, some parents who've actually gone through it themselves.. a parent saying, 'My child never had any side effects.' Or other parents, 'My child did have side effects and this is how we overcame them*.' [Participant 14, mother of vaccinated teenage boy, British African]<br><br>'*If [parents who had their teenagers vaccinated] they can give their views that would be good. 'I gave my child the vaccine, they're still good.' And why they think it's important*.' [Participant 15, parent of unvaccinated teenage girl, Somali]<br><br>'*I think I would prefer to get answers from a medical professional*.' [Participant 25, mother of unvaccinated teenage boys and girls, White British]
	Parents were asked whether they felt faith leaders could advocate for HPV vaccination within the communication materials. Most parents felt it was more appropriate for information to be conveyed by healthcare professionals who had the specialist knowledge	'*We've got trust in both [healthcare professionals and faith leaders], but since this is a health-related matter it would probably be of more benefit if it was coming from somebody that is related to health*.' [Participant 28, mother of unvaccinated teenagers, with translation support]
