# Supplementary material for: Developing films to support vaccine-hesitant, ethnically diverse parents’ decision-making about the human papillomavirus (HPV) vaccine: a codesign study
Source: BMJ Open. 2024 Sep 12;14(9):e079539. doi: 10.1136/bmjopen-2023-079539 (PMC11409246; doi:10.1136/bmjopen-2023-079539)
Supplement: Supplementary file 7 [file bmjopen-2023-079539.reviewer_comments.pdf]

PEER REVIEW HISTORY

BMJ Open publishes all reviews undertaken for accepted manuscripts. Reviewers are asked to complete a checklist review form and are provided with free text boxes to elaborate on their assessment. These free text comments are reproduced below.

ARTICLE DETAILS

**TITLE (PROVISIONAL)**	Developing films to support vaccine-hesitant, ethnically diverse parents' decision-making about the human papillomavirus (HPV) vaccine: A co-design study
**AUTHORS**	Fisher, Harriet; Denford, Sarah; Chantler, Tracey; Audrey, Suzanne; Finn, Adam; Hajinur, Huda; Hickman, Matthew; Mounier-Jack, Sandra; Roderick, Marion; Tucker, Leanne; Yates, Julie; Mohamed, Asha

VERSION 1 - REVIEW

**REVIEWER NAME**	Thilly, Nathalie
**REVIEWER AFFILIATION**	CHRU de Nancy, Plateforme d'Aide à la Recherche Clinique
**REVIEWER CONFLICT OF INTEREST**	None
**DATE REVIEW RETURNED**	21-Sep-2023

**GENERAL COMMENTS**	Thank you very much for the opportunity to review this manuscript. The researchers conducted a co-design study to develop communication materials to support vaccine-hesitant, ethnically diverse parents' decision-making about the HPV vaccine by using an evidence-, theory- and person-based approach. The study provides insights and relevant information for the planning and the development of behavioral change interventions, often under-reported in literature. The manuscript is generally clear and well-written. The research design and the methods are appropriate, the results are quite comprehensive. The following review comments are provided for the authors:<br><br>1- In the title and at the end of the background paragraph (the objectives p6, lines 32 – 46), the authors announce co-designing 'communication materials'. In the rest of the manuscript, we discover that 'communication materials' are 4 films (called the COMMUNICATE films). For better clarity, the same words should be used for the name the intervention tool.<br>2- P6, lines 55-57: "informed written and verbal consent will be obtained …". The use of the future tense seems not appropriate here.<br>3- P6, lines 57: the authors talk about 'workshop', that are not mentioned in the rest of the manuscript.

	4- P7, lines 5-6: Research setting in London and South West of England. The authors should be more precise about the research setting.<br>5- P8, line 3: what do the authors call 'primary and secondary data'? Primary data are from literature and secondary from qualitative interviews?<br>6- P8, literature review (lines 18-26): the authors should at least mention the keywords, database(s), location (studies conducted in England?), and time period (manuscript published since when? And until when?) used for the literature review. Likewise, the process to select key papers should be briefly described.<br>7- P8, qualitative interviews (lines 32-46): The authors describe briefly the qualitative interviews and refer to another paper for more details (ref 16). However, this paper is 'under review', and then not yet published. What if it is not published? The authors should at least mention: the schools and community organization selected (number and location), inclusion criteria and process of selection of parents, the choice of the sample size (what about data saturation?), main characteristics of participants (in the results' paragraph).<br>8- P9, lines 11-12: 'Findings in relation with …'. This sentence should be moved to the paragraph Results – Qualitative interviews (P12).<br>9- P9, line 28: Table 1 (results) should not be mentioned at this stage.<br>10- P10, line 7: the authors mention 'ongoing discussions with key stakeholders'. Who are key stakeholders?<br>11- Supplementary files 2 and 3 are not mentioned in the manuscript.<br>12- P11- Results – literature planning (lines 36-38): a flow chart of the manuscripts selected should be briefly described.<br>13- P12- Results – qualitative interviews (from line 45): the inclusion of 29 parents is a result. The authors should justify this sample size. Has data saturation been reached?<br>14- P14 – lines 52-60: this paragraph should be moved to the 'method section' of the manuscript.<br>15- P15- Feedback from ethnically diverse parents and stakeholders (line 59): this action is not identified as a step of the intervention development stage in the 'method' section<br>16- P18 (lines 45-47): The authors mention as a strength the involvement of the parents from a range of socio-economic and ethnic background. However, socio-economic and ethnic characteristics of participants are not presented in the manuscript.<br>17- P18 (lines 47-49): 'Their involvement maximized the acceptability,…'. This comment should be moderated, as it is just an hypothesis.<br>18- P19 (lines 8-11): 'Although participants were ethnically diverse, not all ethnic groups were represented'. Maybe … but ethnic groups are not presented in the 'Results' section.<br>19- The discussion should address the transferability of the present results to other regions or other countries. What are the lessons learned from this study that can be useful for other researchers who want to develop behavioral change interventions?

**REVIEWER NAME**	Caserotti, Marta

**REVIEWER AFFILIATION**	University of Padua
**REVIEWER CONFLICT OF INTEREST**	No competing interests.
**DATE REVIEW RETURNED**	05-Feb-2024

**GENERAL COMMENTS**	Title: Developing communication materials to support vaccine-hesitant, ethnically diverse parents' decision-making about the human papillomavirus (HPV) vaccine: A co-design study<br>Overall, I found the project very interesting, especially for having considered intervention to increase vaccine adherence in a population that is often underrepresented in research. Especially because tailored communication can help reduce vaccine hesitancy, it is important to have strong and innovative interventions to reduce it.<br>You can find here my main comments:<br>Abstract<br>- The authors talk about supporting behavioral change; however, they did not test their intervention. I strongly suggest testing it to increase the value of the paper.<br>- The authors state that overall participants find the film informative but is not very clear how they assessed it.<br><br>Background<br>- I would suggest considering the vaccination rate against HPV in the area of interest for the article (London and the south-west of England), comparing it to the national rate. This addition could help readers who are not very familiar with vaccination rates in the UK to better understand the value of your project.<br>- I would ask the authors to better explain the importance of reducing vaccine hesitancy in people with different ethnic backgrounds.<br>- Authors choose to have a female parent as a member of the public (LT): why don't you consider having at least a mother and a father? Literature shows differences in vaccine hesitancy considering gender, specifically that female are more hesitant than male (see suggested papers).<br>Kiener, L. M., Schwendener, C. L., Jafflin, K., Meier, A., Reber, N., Maurer, S. S., ... & Tarr, P. E. (2022). Vaccine hesitancy and HPV vaccine uptake among male and female youth in Switzerland: a cross-sectional study. BMJ open, 12(4), e053754.<br>Robertson, E., Reeve, K. S., Niedzwiedz, C. L., Moore, J., Blake, M., Green, M., ... & Benzeval, M. J. (2021). Predictors of COVID-19 vaccine hesitancy in the UK household longitudinal study. Brain, behavior, and immunity, 94, 41-50.<br>Luyten, J., Bruyneel, L., & Van Hoek, A. J. (2019). Assessing vaccine hesitancy in the UK population using a generalized vaccine hesitancy survey instrument. Vaccine, 37(18), 2494-2501.<br><br>Results<br>- In the section "Refinement and translation of the COMMUNICATE films" the authors described some of the feedback received for the films, but to whom the movie was presented for feedback? How many people? Expert (if yes, expert in what?) or non-expert?<br>- In the section "Feedback from ethnically diverse parents and stakeholders" the authors state that the films were informative for ethnically diverse parents and stakeholders. How many of them? How did the authors assess this aspect? Please clarify.

	- As already mentioned, I strongly suggest testing the strength of the intervention, providing results of the efficacy of the films in reducing HPV vaccine hesitancy.<br>I wish the authors the best of luck with their manuscript.

VERSION 1 – AUTHOR RESPONSE

**REVIEWER 1**	
Thank you very much for the opportunity to review this manuscript. The researchers conducted a co-design study to develop communication materials to support vaccine-hesitant, ethnically diverse parents' decision-making about the HPV vaccine by using an evidence-, theory- and person-based approach. The study provides insights and relevant information for the planning and the development of behavioral change interventions, often under-reported in literature. The manuscript is generally clear and well-written. The research design and the methods are appropriate, the results are quite comprehensive.	Thank you for your positive overview of the manuscript and taking time to provide comments.
The following review comments are provided for the authors:<br><br>1. In the title and at the end of the background paragraph (the objectives p6, lines 32 – 46), the authors announce co-designing 'communication materials'. In the rest of the manuscript, we discover that 'communication materials' are 4 films (called the COMMUNICATE films). For better clarity, the same words should be used for the name the intervention tool.	Thank you. Throughout the manuscript we have amended any reference to 'communication materials' to 'COMMUNICATE films' as suggested.
2. P6, lines 55-57: "informed written and verbal consent will be obtained …". The use of the future tense seems not appropriate here.	**Page 6, line 59.** We apologise for this typo. We have now amended this sentence to refer to the past tense.
3. P6, lines 57: the authors talk about 'workshop', that are not mentioned in the rest of the manuscript.	**Page 7, lines 3-5.** We have amended the sentence as follows: '*Informed written or verbal consent was obtained from parents and key professionals prior to participating in an interview,*

	feedback session (including workshops or online meetings) *or filming as part of the study*.'
4. P7, lines 5-6: Research setting in London and South West of England. The authors should be more precise about the research setting.	**Page 7, lines 11-13.** We have amended the sentence as follows to include more precise areas the research was undertaken: '*The research was undertaken in areas of London* ***(Hackney, Tower Hamlets)*** *and South West of England* ***(City of Bristol)…***'
5. P8, line 3: what do the authors call 'primary and secondary data'? Primary data are from literature and secondary from qualitative interviews?	**Page 8, line 20.** That is correct. The sentence has been modified as follows for clarification: **'***The intervention planning stage included collating primary and secondary data through a literature review and qualitative interviews,* ***respectively***.'
6. P8, literature review (lines 18-26): the authors should at least mention the keywords, database(s), location (studies conducted in England?), and time period (manuscript published since when? And until when?) used for the literature review. Likewise, the process to select key papers should be briefly described	**Page 8, lines 34-44.** The literature review was undertaken as recommended by the 'person-based approach'. This was not a systematic review – although we have previously undertaken several relevant systematic reviews in the research area and identified other key recent studies that motivated our study.<br><br>This has been clarified in the methods section.
7. P8, qualitative interviews (lines 32-46): The authors describe briefly the qualitative interviews and refer to another paper for more details (ref 16). However, this paper is 'under review', and then not yet published. What if it is not published?<br><br>The authors should at least mention: the schools and community organization selected (number and location), inclusion criteria and process of selection of parents, the choice of the sample size (what about data saturation?), main characteristics of participants (in the results' paragraph).	The paper has now been published and we have updated the reference accordingly.<br><br>**Page 8, line 59 & Page 13, lines 42-45.** Given the updated publication status of the qualitative study and the already lengthy manuscript we would rather keep the details regarding the qualitative study to a minimum. We have however clarified the study areas here and the main characteristic of the participants in the results paragraph.
8. P9, lines 11-12: 'Findings in relation with …'. This sentence should be moved to the paragraph Results – Qualitative interviews (P12).	**Page 13, lines 40-42.** We have moved the sentence as advised.

9. P9, line 28: Table 1 (results) should not be mentioned at this stage.	**Page 9, line 49.** We have removed the reference to Table 1 as suggested.
10. P10, line 7: the authors mention 'ongoing discussions with key stakeholders'. Who are key stakeholders?	**Page 10, lines 30-34.** We have clarified in the manuscript these include members of school-aged immunisation teams, parents from ethnically diverse backgrounds, community advocates, and academics.
11. Supplementary files 2 and 3 are not mentioned in the manuscript.	**Page 10**. Reference to the Supplementary files 2 and 3 are made on lines 28 and 47, respectively.
12. P11- Results – literature planning (lines 36-38): a flow chart of the manuscripts selected should be briefly described.	**Page 11.** The literature review was undertaken as recommended by the 'person-based approach'. This was not a systematic review – although we have previously undertaken several relevant systematic reviews in the research area and identified other key recent studies that motivated our study.<br><br>Therefore, we do not feel inclusion of a flow chart of the manuscripts to be appropriate in this case.
13. P12- Results – qualitative interviews (from line 45): the inclusion of 29 parents is a result. The authors should justify this sample size. Has data saturation been reached?	**Page 13, line 42.** Although the number of participants of the study is already referred to in the methods section **(page 8, line 53)**, we have now made an additional reference to the number of interview participants within the results section.<br><br>**Page 9, lines 7-9** We confirm within the manuscript that recruitment continued until no new themes were identified during the interviews.
14. P14 – lines 52-60: this paragraph should be moved to the 'method section' of the manuscript.	**Page 11, paragraph 3**. The paragraph has been moved as suggested.
15. P15- Feedback from ethnically diverse parents and stakeholders (line 59): this action is not identified as a step of the intervention development stage in the 'method' section	**Page 11.** This phase is described in the following section 'Refinement and translation of the COMMUNICATE films'.<br><br>**Page 16, line 20.** For clarity, we have amended the sub-heading to '***Summary of*** *feedback from ethnically diverse parents and stakeholders'.*
16. P18 (lines 45-47): The authors mention as a strength the involvement of the parents from a	**Page 13, lines 42-45, Table 2, Page 16, lines 15-21, and Page 17, lines 22-26.** The ethnicities of

range of socio-economic and ethnic background. However, socio-economic and ethnic characteristics of participants are not presented in the manuscript.	interview participants, film participants, and feedback contributors are now provided.<br><br>**Page 20, line 26.** We have removed reference to sociodemographic characteristics as this was not formally collected.
17. P18 (lines 47-49): 'Their involvement maximized the acceptability,…'. This comment should be moderated, as it is just an hypothesis.	**Page 20, line 28.** We have amended the sentence as follows: '*Their involvement **may help improve** the acceptability, feasibility, and persuasiveness of the COMMUNICATE films*.'
18. P19 (lines 8-11): 'Although participants were ethnically diverse, not all ethnic groups were represented'. Maybe … but ethnic groups are not presented in the 'Results' section.	**Page 13, lines 42-45, Table 2, Page 16, lines 15-21, and Page 17, lines 22-26.** The ethnicities of interview participants, film participants, and feedback contributors are now provided.
19. The discussion should address the transferability of the present results to other regions or other countries.<br><br>What are the lessons learned from this study that can be useful for other researchers who want to develop behavioral change interventions?	**Page 18, lines 41-43.** We have added the following sentence within the Discussion section; **'***Although developed in two regions, the COMMUNICATE films are likely to be relevant to other ethnically diverse communities in other regions of England***.'**<br><br>**Page 19, paragraph 3**. We have expanded the section included under the sub-heading 'Key learning' within the Discussion section.
**REVIEWER 2**	
Overall, I found the project very interesting, especially for having considered intervention to increase vaccine adherence in a population that is often underrepresented in research. Especially because tailored communication can help reduce vaccine hesitancy, it is important to have strong and innovative interventions to reduce it.	Thank you for your interest in our manuscript.
Abstract<br><br>- The authors talk about supporting behavioral change; however, they did not test their intervention. I strongly suggest testing it to increase the value of the paper.	We agree with the comment that testing interventions is important to establish effectiveness and unintended consequences. However, the purpose of this manuscript was to report an earlier stage in the evaluation process: intervention development.
- The authors state that overall participants find the film informative but is not very clear how they assessed it.	**Abstract & Page 17, lines 30-32**. We have amended the sentence as follows: 'Overall, parents and stakeholders **were positive about the films.'**

Background<br><br>- I would suggest considering the vaccination rate against HPV in the area of interest for the article (London and the south-west of England), comparing it to the national rate. This addition could help readers who are not very familiar with vaccination rates in the UK to better understand the value of your project.	**Page 7, paragraph 2.** We have now included the vaccination rates in the local authorities where the research activities were undertaken: '*The research was undertaken in areas of London (Hackney, Tower Hamlets) and South West of England (City of Bristol), where uptake rates of the HPV vaccination programme are ranked below the national average of 69.6% of the cohort receiving the first dose (61.8% and 68.5%, respectively).*'
- I would ask the authors to better explain the importance of reducing vaccine hesitancy in people with different ethnic backgrounds.	**Page 5, lines 38-40**. We have amended the following sentence which references evidence specific to ethnic minority groups as follows: '*Vaccine hesitancy is recognised as an important contributor to inequalities in uptake of vaccination programmes* ***among minority ethnic groups*** *[7, 8].*'
- Authors choose to have a female parent as a member of the public (LT): why don't you consider having at least a mother and a father? Literature shows differences in vaccine hesitancy considering gender, specifically that female are more hesitant than male (see suggested papers).<br><br>Kiener, L. M., Schwendener, C. L., Jafflin, K., Meier, A., Reber, N., Maurer, S. S., ... & Tarr, P. E. (2022). Vaccine hesitancy and HPV vaccine uptake among male and female youth in Switzerland: a cross-sectional study. BMJ open, 12(4), e053754.<br><br>Robertson, E., Reeve, K. S., Niedzwiedz, C. L., Moore, J., Blake, M., Green, M., ... & Benzeval, M. J. (2021). Predictors of COVID-19 vaccine hesitancy in the UK household longitudinal study. Brain, behavior, and immunity, 94, 41-50.<br><br>Luyten, J., Bruyneel, L., & Van Hoek, A. J. (2019). Assessing vaccine hesitancy in the UK population using a generalized vaccine hesitancy survey instrument. Vaccine, 37(18), 2494-2501.	Our public contributor became involved in the project because of an ongoing relationship with a member of the research team and her interest in contributing to the project, rather than being selected because of her gender. We will consider ensuing fathers' perspectives are included in future projects.
Results<br><br>- In the section "Refinement and translation of the COMMUNICATE films" the authors described some of the feedback received for the films, but to whom the movie was presented for feedback? How many people? Expert (if yes, expert in what?) or non-expert?	**Page 17, lines 22-26.** We have clarified the number of parents and stakeholders contributors with their area of expertise within this section of the manuscript.

- In the section “Feedback from ethnically diverse parents and stakeholders” the authors state that the films were informative for ethnically diverse parents and stakeholders. How many of them? How did the authors assess this aspect? Please clarify.	**Page 17, lines 22-26.** We have clarified the number of participants and their ethnicities within this section of the manuscript.
- As already mentioned, I strongly suggest testing the strength of the intervention, providing results of the efficacy of the films in reducing HPV vaccine hesitancy.	As described previously, we agree that testing interventions is important to establish effectiveness and unintended consequences. However, the purpose of this manuscript was to report an earlier stage in the evaluation process: intervention development.
I wish the authors the best of luck with their manuscript.	Thank you.

VERSION 2 - REVIEW

**REVIEWER NAME**	Caserotti, Marta
**REVIEWER AFFILIATION**	University of Padua
**REVIEWER CONFLICT OF INTEREST**	None
**DATE REVIEW RETURNED**	16-Apr-2024

**GENERAL COMMENTS**	I have reviewed the paper " Developing films to support vaccine-hesitant, ethnically diverse parents’ decision-making about the human papillomavirus (HPV) vaccine: A co-design study ," and I really liked the ambition of the project.<br><br>In general, I appreciated the intervention proposed by the authors, which is the result of a systematic, theory- and people-based approach. Nevertheless, I believe the contribution, if accompanied by data on the effectiveness of the proposed intervention, could be much more relevant in efforts to counter vaccine hesitancy.<br>The theoretical background, in my opinion, could benefit from a better framing of the problem of vaccine hesitancy in ethnic minority groups, emphasizing the importance of ad hoc interventions to counter vaccine misinformation and to promote immunization adherence.

VERSION 2 – AUTHOR RESPONSE

**REVIEWER 2**	
In general, I appreciated the intervention proposed by the authors, which is the result of a systematic, theory- and people-based approach. Nevertheless, I believe the contribution, if	We agree with the comment that testing interventions is important to establish effectiveness and unintended consequences. However, the purpose of this manuscript was to

accompanied by data on the effectiveness of the proposed intervention, could be much more relevant in efforts to counter vaccine hesitancy.	report an earlier stage in the evaluation process: intervention development. We currently do not have evidence related to it's effectiveness but have a future project planned to develop and evaluate the implementation of a communication strategy with the films.
The theoretical background, in my opinion, could benefit from a better framing of the problem of vaccine hesitancy in ethnic minority groups, emphasizing the importance of ad hoc interventions to counter vaccine misinformation and to promote immunization adherence.	**Page 5, paragraph 4:** We have expanded our framing of vaccine hesitancy among minority ethnic groups within the Introduction section as follows:<br><br>*'Vaccine hesitancy is recognised as an important contributor to inequalities in uptake of vaccination programmes among minority ethnic groups [7, 8].* ***Contributors to higher vaccine hesitancy among minority ethnic groups include concerns relating to safety and side effects of vaccines, a lack of trust in the medical profession due to historical discrimination, racist ideology and immoral experimentation on specific racial groups [8-11]. Meaningful engagement and tailored information can support effective vaccine promotion. Engagement and knowledge are facilitated by collaboration with trusted community representatives.*** *To address inequalities in vaccine hesitancy,* ***community-led strategies are required that incorporate meaningful engagement to ensure diverse local voices are heard and codesign programmes that address local concerns to maximise vaccine uptake from the ground up [12]. This includes*** *health communication interventions that take into account the cultural and community contexts that influence beliefs and decisions about vaccines among different groups [13].'*
